# Computational Framework for Prediction of Peptide Sequences That May Mediate Multiple Protein Interactions in Cancer-Associated Hub Proteins

**DOI:** 10.1371/journal.pone.0155911

**Published:** 2016-05-24

**Authors:** Debasree Sarkar, Piya Patra, Abhirupa Ghosh, Sudipto Saha

**Affiliations:** 1 Bioinformatics Centre, Bose Institute, Kolkata, India; 2 Maulana Abdul Kalam Azad University of Technology, Kolkata, India; University of Erlangen-Nuremberg, GERMANY

## Abstract

A considerable proportion of protein-protein interactions (PPIs) in the cell are estimated to be mediated by very short peptide segments that approximately conform to specific sequence patterns known as linear motifs (LMs), often present in the disordered regions in the eukaryotic proteins. These peptides have been found to interact with low affinity and are able bind to multiple interactors, thus playing an important role in the PPI networks involving date hubs. In this work, PPI data and de novo motif identification based method (MEME) were used to identify such peptides in three cancer-associated hub proteins—MYC, APC and MDM2. The peptides corresponding to the significant LMs identified for each hub protein were aligned, the overlapping regions across these peptides being termed as overlapping linear peptides (OLPs). These OLPs were thus predicted to be responsible for multiple PPIs of the corresponding hub proteins and a scoring system was developed to rank them. We predicted six OLPs in MYC and five OLPs in MDM2 that scored higher than OLP predictions from randomly generated protein sets. Two OLP sequences from the C-terminal of MYC were predicted to bind with FBXW7, component of an E3 ubiquitin-protein ligase complex involved in proteasomal degradation of MYC. Similarly, we identified peptides in the C-terminal of MDM2 interacting with FKBP3, which has a specific role in auto-ubiquitinylation of MDM2. The peptide sequences predicted in MYC and MDM2 look promising for designing orthosteric inhibitors against possible disease-associated PPIs. Since these OLPs can interact with other proteins as well, these inhibitors should be specific to the targeted interactor to prevent undesired side-effects. This computational framework has been designed to predict and rank the peptide regions that may mediate multiple PPIs and can be applied to other disease-associated date hub proteins for prediction of novel therapeutic targets of small molecule PPI modulators.

## Introduction

There has been a gradual shift of focus in cancer research from the study of individual proteins to edgetic perturbations of highly connected nodes (proteins) in intra-cellular signaling networks, known as hub nodes, which are considered essential for maintaining the network topology [1–3]. Hubs that directly interact with most or all of their partners simultaneously are called 'party' hubs (multi-interface hubs), whereas those that bind different partners at different times or locations are known as 'date' hubs (singlish-interface hubs) [4]. A growing number of protein-protein interactions (PPIs) are now known to be mediated by short linear peptides, where a globular protein or domain binds to short peptide segments in multiple partners, generally located in the intrinsically disordered regions [5,6]. Such peptides may sometimes be present in ordered segments also, e.g. the p53 peptide that binds to MDM2 occurs in ordered helical region [7]. These peptide segments may occur in different regions of the interacting proteins, but sequence analysis often reveals an underlying consensus pattern or linear motif (LM) that captures the key structural and physicochemical features of the regions [8]. The small peptides have been shown to mimic the protein-protein interactions and may thus be useful in extracting interacting partners in experimental procedures like affinity purification [9]. The transient and low-affinity PPIs mediated by these short, flexible peptide segments help many date hub proteins to employ the same interfaces for binding multiple interactors at different time or locations [10,11]. Furthermore, mutations in such peptide sequences of signaling hub proteins may affect entire PPI networks and signaling cascades [12]. Recent studies have shown that small chemical inhibitors can target PPIs, including the ones mediated by short peptides, and have the potential to act as new therapeutic agents against complex diseases including cancer [13]. Therefore, identification of such short peptides that may mediate multiple protein interactions in essential cancer-associated hub proteins can help in targeting peptide-mediated PPIs for therapeutic intervention with structural analogues.

**Fig 1:**
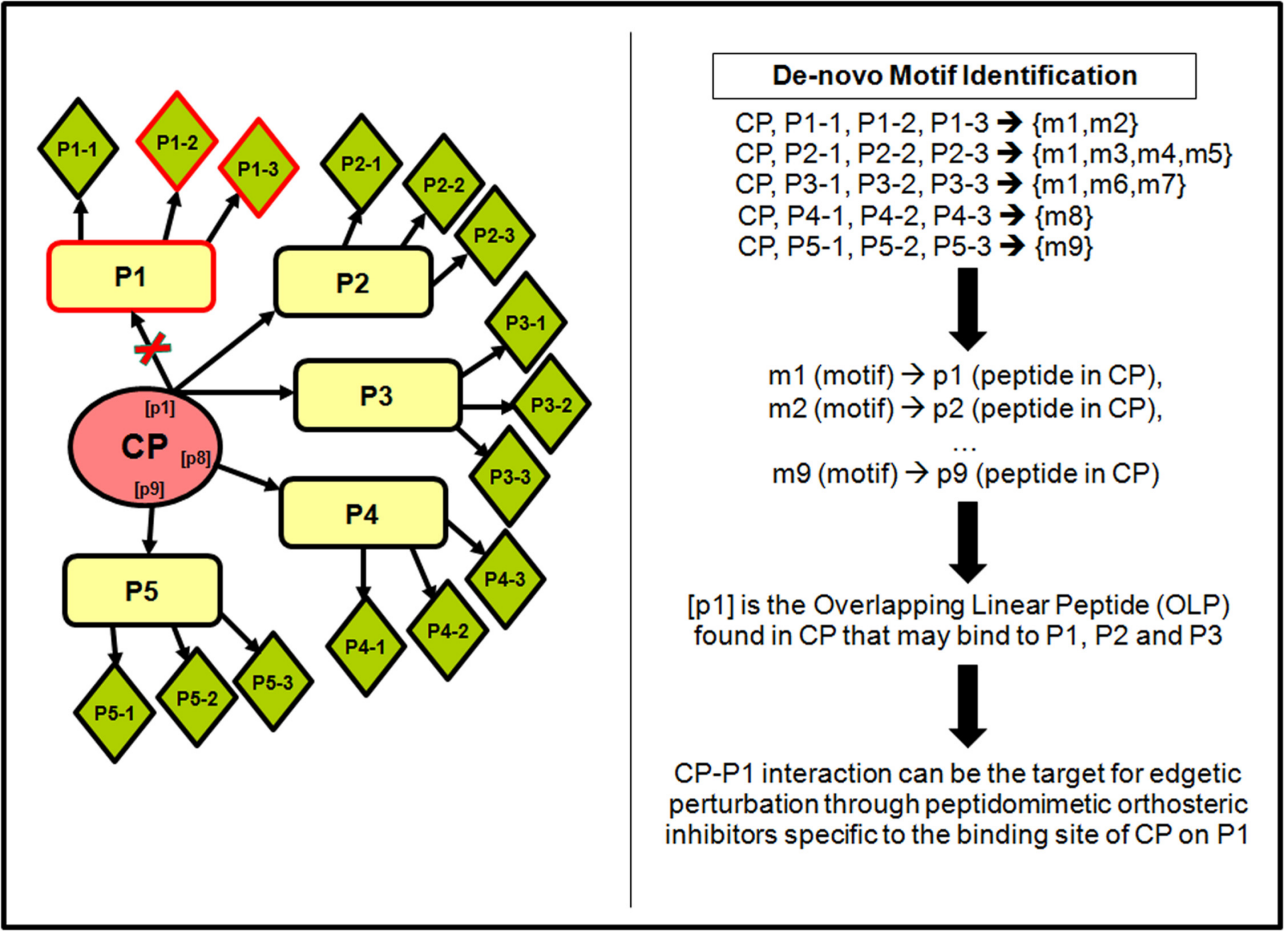
CP represents a multifunctional cancer-associated hub protein and P1, P2, P3, P4 & P5 are its direct interactors (First Hop Protein Interactors or FHPIs of CP). P1-1, P1-2 & P1-3 are the interactors of P1 (Second Hop Protein Interactors or SHPIs of CP), P2-1, P2-2 & P2-3 are the interactors of P2 and so on. Red-borders have been used to mark the oncoproteins. Sequence analysis (using MEME for de-novo motif identification) of all the interactors of a particular FHPI (e.g CP, P1-1, P1-2, and P1-3 for P1) may reveal some shared sequence patterns (e.g. m1 & m2 among interactors of P1, m1, m2, m3 & m4 among interactors of P2 etc). Alignment of the peptide sequences from CP corresponding to all such motifs (p1 from m1, p2 from m2 etc) may then identify a common peptide (OLP) from the overlapping sequence positions. This OLP may play a key role in mediating interactions with multiple FHPIs and therefore help in designing orthosteric inhibitors that can be targeted to block any of the CP-FHPI interactions by making it specific to the binding site of CP on a particular FHPI (e.g. P1).

The goal of the present study is to develop a computational framework for predicting peptide sequences in cancer-associated hub proteins (CPs) that may bind to multiple interactors, using experimentally verified PPI datasets and a network-based approach. In a protein interaction network, where the nodes represent the proteins and the edges their mutual interactions, most of the nodes are not directly connected to one another, but any of the nodes can be reached from any other node in the network through a small number of hops or edges. The first hop protein interactors or FHPIs (the yellow rectangles marked as P1, P2… P5 in **Fig 1**) are the ones directly connected to CP (the pink oval central node) by edges (black arrows). The second hop protein interactors or SHPIs are those that are connected to the CP through the FHPIs (the green rhomboids viz. P1-1, P1-2 & P1-3 through P1; P2-1, P2-2 & P2-3 through P2 etc in **Fig 1**) [14]. We have chosen three well-known cancer-associated human hub proteins viz. MYC, APC and MDM2, each known to be linked to a large number of FHPIs and a proportionately larger number of SHPIs. The interaction networks of these three proteins were reconstructed up to the second hop level by gathering the list of FHPIs interacting with each of the CPs, followed by the list of SHPIs interacting with each of the FHPIs.

MEME (Multiple Em for Motif Elicitation) [15,16] is a very popular and widely used tool for searching ungapped sequence patterns repeated across a set of fasta sequences. The amino-acid sequences of all the interactors of an FHPI (i.e., SHPIs as well as the CP itself), were submitted to MEME for identification of the over-represented sequence patterns (LMs) present in all or most of these proteins, which may mediate interactions with the FHPI. We hypothesized that since the FHPI is a common interactor for the set of corresponding SHPIs and the CP, some of these sequences may share a motif denoting the peptide regions interacting with the FHPI. The MEME analysis for motif identification was repeated for every FHPI of a CP independently, to compile a list of motifs, each of which was predicted to interact with a particular FHPI of a CP. After compiling the list of FHPI-specific MEME-predicted motifs, we focused on the peptide segments in CP that correspond to these patterns in the MEME results. Our aim is to align these peptides and reveal the overlapping sequence positions among them, which may be predicted as the peptide interfaces that may interact with multiple FHPIs [17,18]. These overlaps have been denoted henceforth as Overlapping Linear Peptides or OLPs.

Our proposed workflow is an attempt to provide a coarse-grained prediction of the regions within singlish-interface hubs that may mediate multiple PPIs, using existing in-silico methods for motif elucidation, thereby facilitating further experimental studies on them. MEME was chosen for the motif identification step because it does not adjust for evolutionary relationships, (unlike DILIMOT[19], SLiMDisc[20], SLiMFinder[21] and QSLiMFinder[22]) therefore accounting for sequence patterns responsible for PPIs in evolutionarily related proteins [23]. A scoring system was also formulated to rank the predicted OLPs according to a new metric designated as OLP score, normalized across all three CPs by comparing with the median OLP score from randomly generated sets of protein sequences. For validating the proposed workflow, we repeated the procedure with the interaction network of human GASP2, which involves at least three experimentally verified examples of a single peptide mediating multiple PPIs [24]. We also made an attempt to evaluate the predicted OLP-mediated FHPI interactions through the PepSite2 [25] web server, which predicted that several OLPs from MYC and MDM2 can bind to multiple FHPIs. Furthermore, we also performed a BLAST search with the predicted OLP sequences to find similar peptide sequences in human proteins not present in the SHPI networks used in our study, to enable prediction of novel PPIs.

## Materials and Methods

### Overview of the proposed workflow

The first task is to recreate the PPI network of the hub protein (CP) up to the second hop level by compiling the list of proteins known to interact with the CP (FHPI network) and then the proteins interacting with each of the FHPIs (SHPI network). In **Fig 1**, the nodes in the FHPI network have been shown as yellow rectangles and the nodes in the SHPI network as green rhomboids. In the next step, interactors of each FHPI (including the CP) were scanned for shared sequence patterns using MEME. For example, let us suppose protein P1 is known to directly interact with CP, thus being the FHPI of CP. P1 is also known to interact with three other proteins P1-1, P1-2 & P1-3, which would be SHPIs of CP. The sequences of CP, P1-1, P1-2 and P1-3 are submitted together to MEME for finding shared sequence patterns among them and two such patterns m1 and m2 are found. Here we have hypothesized that since the four proteins have a common interactor (i.e. P1), the motifs shared by them may mediate their interactions with P1. The same process is repeated with P2, the next FHPI, and MEME analysis of its interactors i.e., CP, P2-1, P2-2, and P2-3, show the shared sequence patterns m1, m3, m4 and m5. Hence, these motifs may be predicted to mediate interactions with P2. Similarly, other motifs are predicted for each of the remaining FHPIs, P3, P4 and P5. The peptide sequences of CP that correspond to the motifs (say p1 corresponds to m1, p2 to m2 etc) are then compared to see if some of them overlap. If such overlaps are found, then these overlapping positions may be hypothesized to mediate interactions with multiple FHPIs (e.g. p1 may be predicted to interact with P1, P2 and P3).

### Protein-Protein Interaction Dataset

Three cancer-associated hub proteins- MYC, APC and MDM2, were used in the study for identifying LMs, and were found to be associated with 721, 95, 177 FHPIs and 4850, 1000, 3047 SHPIs respectively, according to the IntAct database [26] (**Table A in S1 File**). Only experimentally verified human protein-protein interactions were considered in this study.

### Amino acid sequences of proteins

The amino acid sequences of the CPs (MYC, APC and MDM2) and the other SHPIs were extracted from the UniProt database [27] in fasta format.

### Motif identification

The protein sequences of the direct interactors of each FHPI i.e. the CP and other SHPIs, (e.g CP, P1-1, P1-2, and P1-3 for P1), were used for de novo motif identification by MEME. The E-value in the MEME output was used to infer the statistical significance of each of the reported sequence patterns or LMs, whereas, the p-value was used to determine the extent of matching of individual peptide instances to the corresponding LM. The statistically significant (E-value<1.0) motifs observed in the CP as well as in other SHPIs, were selected for further analysis. The FHPIs with 5–20 interactors were only used in this study because with increase of sequence variability across multiple interactors, it becomes difficult to identify conserved regions among them using de-novo motif identification. An E-value cut-off of 1.00 was chosen for a higher sensitivity at the cost of lower specificity [28]. The standalone version of MEME was used with parameters set to 'zoops' (zero or one per sequence) for distribution of motifs, 6 as minimum and 50 as maximum motif width, and 10 as maximum number of motifs to be reported.

### Multiple Sequence Alignment of Motifs

The peptide sequences in the CP corresponding to the significant motifs identified from multiple MEME runs were aligned using Clustal Omega [29], followed by manual interpretation of the alignments to find possible overlaps among them, thereby reducing the probability of reporting false positives.

### OLP Score

The short overlapped peptide sequences identified in each of the CPs (MYC, APC and MDM2) were ranked according to the **OLP score**^**observed**^ computed as:

$${\mathrm{OLP score}}^{\mathrm{observed}}=log\;(NFP/(TFP*\text{HM}))$$

Where NFP and TFP represent the number of FHPIs interacting with an OLP and the total number of FHPIs of a CP respectively, whereas HM denotes the Harmonic Mean of the p-values reported by MEME for all the longer peptides having the OLP.

### Generation of OLP scores from random PPI networks

Twenty-five decoy protein interaction networks were generated by grouping random numbers of protein sequences chosen randomly from a dataset comprising of the fasta sequences of the entire human proteome set from UniProt [27]. OLP scores were generated for these 25 sets treating them as 25 FHPI networks and the distribution of these scores were plotted using R scripts. This procedure was repeated four times, creating 25 X 4 = 100 decoy FHPI networks, and the four separately plotted distributions showed median values of 15.42, 20.03, 17.4 and 18.25 respectively (**Fig A (i), (ii), (iii) & (iv) in S1 File**). The average of the median values from the four distributions was 17.78 with a standard deviation of 1.91. Hence we assumed **OLP score**^**random**^ **= 17.78**. The **OLP score**^**observed**^ was normalized by dividing with the **OLP score**^**random**^ giving the **OLP score**^**normalized**^:

$$OLP_{score}{}^{normalized}=OLP_{score}{}^{observed}/OLP_{score}{}^{random}$$

### Validation using multiple orthogonal approaches

#### i) Structural bioinformatics

The PepSite2 [25] server was used to analyze the interactions between each of the OLPs with the respective FHPIs. Pepsite2 predicts the binding site for peptides on protein surfaces using spatial position specific scoring matrices (S-PSSMs) calculated from known 3D structures of protein-peptide complexes derived from PDB. Users need to provide the query peptide sequence in standard one-letter amino acid codes, as well as the structure of the peptide-binding protein in the PDB format. Since the structures of some FHPIs used in this study were not available in the PDB database, their modeled structures were either obtained from ModBase[30] (if available), or modeled using the SWISS-MODEL [31] homology-modeling server. The details of PDB structures chosen as templates for modeling FHPI structures were reported in **Table E in S1 File**.

#### ii) Functional grouping analyses

The gene ontology enrichment and pathway analyses (Reactome and KEGG) of the CP and the FHPIs interacting with each of the identified OLPs were performed using ClueGO [32] plug-in of Cytoscape (k-value set at 0.3) and FuncAssociate 2 [33].

#### iii) Other analyses

Disordered region of the CP were searched in DisProt [34], IUpred [35] and ESpritz [36] and the locations of the identified OLPs were mapped accordingly. In addition, the protein sequences of the CPs (MYC, APC and MDM2) were scanned using the ELM [37] resource and searched in the LMPID [38] database, to look for already reported linear motif instances that coincide with those identified in this study. Surface accessibility of the OLP sequences was verified by submitting the CP sequences to SCRATCH prediction server [39].

#### iv) Validation of the proposed workflow using known example of peptide mediating multiple PPIs

GASP2_HUMAN (Q96D09) was assumed to be a CP and three human proteins- ADRB1, ACM1 and CALCR, known to bind to the same peptide of GASP2, were assumed to be the FHPIs, in this study. The human proteins interacting with each of these three FHPIs were listed from the IntAct database [26], which form the SHPI sets. The fasta sequences of the SHPIs for each FHPI were submitted to MEME along with the sequence of the CP (i.e. GASP2_HUMAN). The significant motifs reported by MEME were checked for peptides from GASP2 and all such peptides were aligned to search for overlapping positions.

### BLAST homology search with predicted OLPs

The OLP sequences were searched against the human protein sequences in SwissProt database using protein BLAST, to identify possible sequence matches that may indicate possible interactions between these proteins and the respective FHPIs.

## Results

### OLPs found in MYC protein

**Fig 2:**
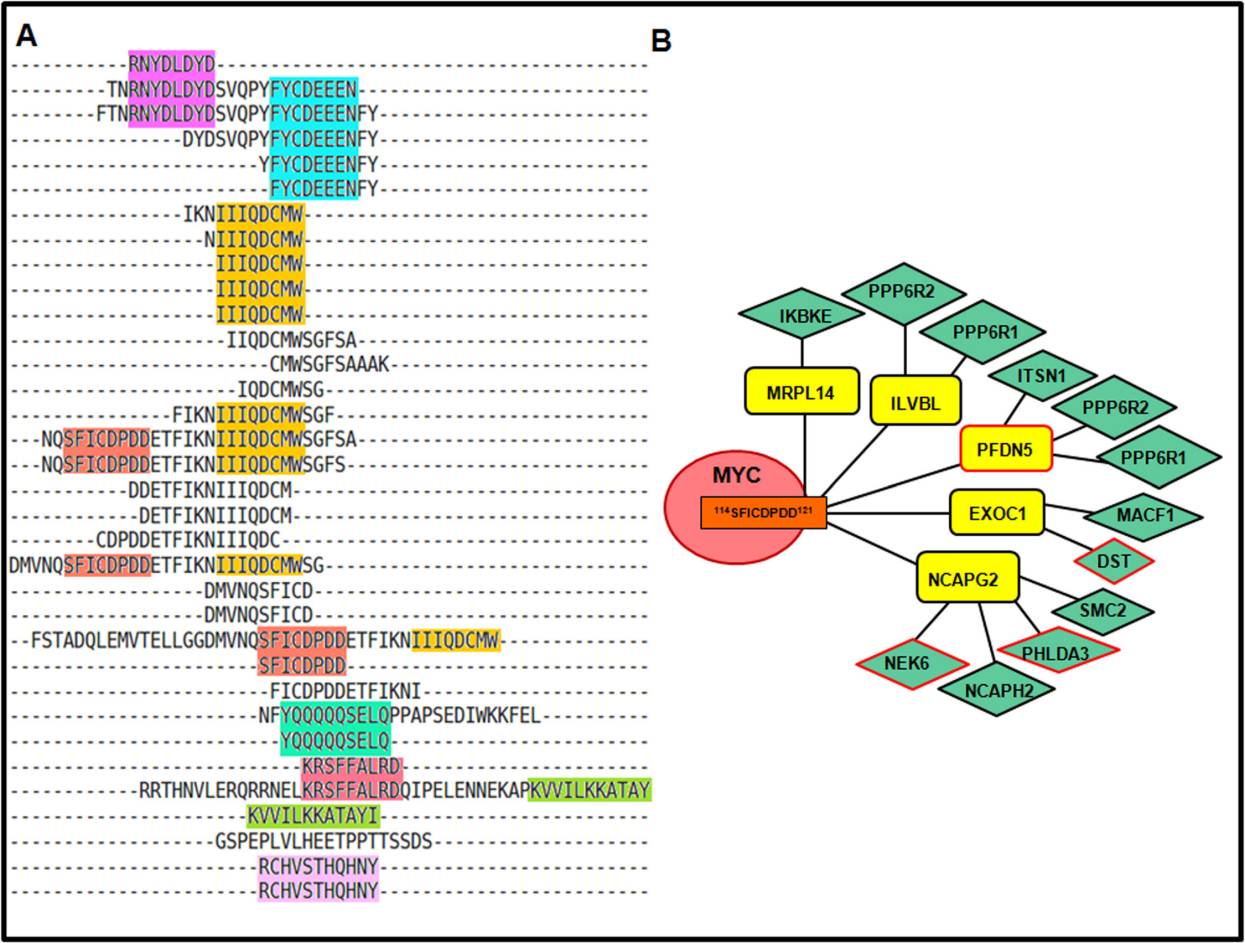
(A) Eight OLP sequences (each marked by a different background colour) were identified by Multiple Sequence Alignment of the peptide sequences in MYC corresponding to all significant motifs (E-value<1.0) inferred by MEME from its SHPI network. (B) Diagrammatic representation of an OLP sequence ^114^SFICDPDD^121^ (marked with orange background) identified in MYC, which may interact with five FHPIs. The nodes representing oncoproteins have been marked with a red border.

MYC (439 aa) is a multifunctional transcriptional factor and an important signaling hub. A mutated form of c-MYC is found in many human cancers, including lung cancer, breast cancer, cervical cancer, ovarian cancer, and in colon and colorectal cancer, where Myc is constitutively expressed [40,41]. In MEME runs, only 292 FHPIs with degree in between 5–20 (**Fig B in S1 File**) were used, and 34 significant motifs were reported in MYC by MEME outputs (**Table B in S1 File**), from which 8 OLPs were identified, as shown in **Fig 2 (A)** and **Table 1**. The normalized OLP scores of 6 out of the 8 OLPs were greater than 1 (indicating these scores were better than OLP scores for random PPI networks). For example, the C-terminal OLPs of MYC, ^371^KRSFFALRD^379^ and ^392^KVVILKKATAY^402^, were shown to be involved in NOTCH1 transcriptional process (Reactome) and cellular response to UV (enriched GO BP). These two peptides were also predicted to interact with FBXW7 that forms a part of the E3 ubiquitin-protein ligase complex, and thus can mediate proteasomal degradation of MYC. However only the sequence ^371^KRSFFALRD^379^ was predicted to lie in the disordered region and be partly surface accessible (**Fig I (i) in S1 File**), and thus may be expected to mediate multiple PPIs. PepSite2 also predicted the binding of this peptide to two FHPIs—CNOT4 and FBXW7, with reasonably significant p-values (**Table F** and **Fig H1-2 in S1 File**). Similarly, the N-terminal OLMs, ^10^RNYDLDYD^17^ and ^23^FYCDEEEN^30^, were predicted to have a role in E-box binding based on enriched GO molecular functions terms. Both these peptides lie in disordered regions, are surface accessible (**Fig I (i) in S1 File**), and were predicted by the ELM resource to contain motif instances of the 'LIG' category. Another OLP sequence of MYC, ^114^SFICDPDD^121^, with a normalized OLP Score of 1.76, predicted to lie in disordered region and be surface accessible (**Fig I (i) in S1 File**), has been shown in **Fig 2 (B)**. This OLP may interact with five FHPIs viz. EXOC1, ILVBL, PFDN5, NCAPG2 and MRPL14. However, no common functional annotations (GO terms or pathways) were found to be associated with these five proteins. This may indicate that these FHPIs could interact with the same OLP of MYC but at different time or locations.

**Table 1:** Overlapping Linear Motifs (OLPs) found in the cancer-associated hub proteins (CPs) MYC, APC and MDM2.

Name of the CP	OLP sequence	OLP score	Name of the FHPIs	SecStr*	Surface Accessible	Enriched GO terms / Pathways	ELM prediction
MYC	371-KRSFFALRD-379	2.95	CNOT4, FBXW7	D	Partly	**NOTCH1 Intracellular DomainRegulates Transcription** (REACTOME);**Cellular response to UV** (GO_BP)	**_**
MYC	114-SFICDPDD-121	1.76	EXOC1, ILVBL, PFDN5,NCAPG2, MRPL14	D	Yes	-	**_**
MYC	128-IIIQDCMW-135	1.76	EXOC1, RAB11FIP5, BPTF, ILVBL, PFDN5, MSH3, MRPL14, NUP188, ZCCHC11, KPNA4	D	No	-	**_**
MYC	32-YQQQQQSELQ-41	0.92	KIF20B, KALRN	D	Yes	-	LIG_SH2_STAT3
MYC	392-KVVILKKATAY-402	2.95	FBXW7, TCF12	H	No	**NOTCH1 Intracellular Domain Regulates Transcription** (REACTOME); **Cellular response to UV** (GO_BP); **E-box binding** (GO_MF)	**_**
MYC	23-FYCDEEEN-30	1.47	MSH3, GIGYF2, FASTKD2, NFIL3, TCF12	D	Yes	**E-box binding** (GO_MF)	LIG_SH2_STAT5
MYC	10-RNYDLDYD-17	1.47	FASTKD2, IL4R, TCF12	D	Partly	**E-box binding** (GO_MF)	LIG_TYR_ITIM
MYC	299-RCHVSTHQHNY-309	0.56	NCAPG2, MYO1B	D	Yes	-	LIG_14-3-3_3
APC	416-YCETCWEW-423	0.76	GIGYF2, EPAS1, ANKRD17	H	No	**Anatomical structure homeostasis** (GO_BP)	LIG_SH2_STAT5
APC	422-EWQEAH-427	0.76	NCKAP5, ANKRD17	H	Yes	-	_
APC	155-KDWYYA-160	0.35	CYTH2, GIGYF2	H	Yes	-	_
MDM2	456-GHLMACF-462	1.60	RNF8, GLTSCR2, TSNAX, FKBP3	H	No	-	_
MDM2	463-TCAKKLKKRNKPC-475	1.60	RNF8, HLA-DMB, FKBP3, JUND	H	Yes	-	_
MDM2	475-CPVCR-478	0.88	PHF7, HLA-DMB, JUND	C	Yes	-	_
MDM2	305-CTSCN-309	1.95	HRSP12, MAP4K4, PIM2, ARHGEF6, NEFM, ZNF326, TSNAX	C	Yes	-	_
MDM2	311-MNPPLPSHC-319	1.95	MAP4K4, PIM1, PIM2, ARHGEF6, YY1AP1, NEFM, ZNF326, TSNAX, USP2	C	Partly	**Positive regulation of cell cycle phase transition** (GO_BP);**Positive regulation of mitotic cell cycle phase transition** (GO_BP); **Positive regulation of G1/S transition of mitotic cell cycle** (GO_BP); **Positive regulation of mitotic cell cycle** (GO_BP)	LIG_SH3_3 LIG_WW_2DOC_USP7_1
MDM2	438-CVICQ-442	1.60	PHF7, TSNAX, FKBP3	E	Yes	-	_

Each row represents the name of the hub protein, the sequence of the OLP, the OLP score^normalized^, name of FHPIs that may interact with the OLP, secondary structure of the OLP, whether the OLP is surface accessible, enriched GO terms & pathways among the FHPIs and the CP, and presence of the OLP in the ELM resource.

*Secondary Structure: H-Helical, E-Sheet, C-Coil, D-Disordered.

### OLPs found in APC protein

Adenomatous polyposis coli (APC) protein (2843 aa) is a large multi-domain protein encoded by the tumor suppressor APC gene, and is involved in the Wnt signaling pathway that plays an integral role in cell adhesion and proliferation in cancer [42]. There were 95 FHPIs and 1000 SHPIs of APC as reported in IntAct [25], out of which there were 40 FHPIs (degree in between 5–20) used in our analysis (**Fig C in S1 File**). Only 8 significant motifs were reported by MEME runs (**Table C in S1 File**), out of which 3 OLPs could be identified, as shown in **Fig E in S1 File** and **Table 1**. These 3 OLPs were all located within the Armadillo-like helical domain (ordered region), and none of them scored higher than the median OLP score from random PPI networks. Hence, we could not consider these OLPs as peptides that may mediate multiple PPIs, according to our framework. This is also reflected in the PepSite2 docking studies where none of the OLPs from APC showed appreciable binding to the respective FHPIs (**Table F** and **Fig H28-34 in S1 File**). It is quite possible that due to its increased length as compared to the other two CPs considered in this study, APC may not need to employ single peptide interfaces for multiple PPIs.

### OLPs found in MDM2 protein

MDM2 (491 aa) is the E3 ubiquitin-protein ligase that mediates ubiquitination of the p53 tumour suppressor [43]. There are 177 FHPIs and 3047 SHPIs of MDM2 as reported in IntAct [26] database, out of which 68 FHPIs (degree in between 5–20) were used in our study (**Fig D in S1 File**). MEME runs predicted 18 significant motifs (**Table D in S1 File**), from which 6 OLPs were identified, out of which 5 OLPs have higher score than the random OLP score (**Fig F in S1 File** and **Table 1**). We have predicted three peptides in the region from 438–475 of MDM2 (^438^CVICQ^442^, ^456^GHLMACF^462^ and ^463^TCAKKLKKRNKPC^475^) that can bind to FKBP3 (also known as FKBP25), which regulates the p53-MDM2 pathway. PepSite2 also predicts the binding of FKBP3 to both the OLPs ^438^CVICQ^442^ (**Fig H60 in S1 File**) and ^456^GHLMACF^462^ (**Fig H37 in S1 File**) as highly significant and to ^463^TCAKKLKKRNKPC^475^ (**Fig H40 in S1 File**) as moderately significant (**Table F in S1 File**). However, only ^438^CVICQ^442^ and ^463^TCAKKLKKRNKPC^475^ were predicted to be surface accessible (**Fig I (iii) in S1 File**). The C-terminal OLP ^311^MNPPLPSHC^319^ may interact with nine FHPIs, which are associated with positive regulation of cell cycle and regulation of protein stability. In addition, this OLP comprises of two ELM predicted motif instances– ^311^MNPPLP^316^ (LIG_SH3_3) and ^313^PPLP^316^ (LIG_WW_2). PepSite2 predicts the binding of this OLM with six FHPIs, out of which for three FHPIs the binding is highly significant. Interestingly, the p-value for binding of this SH3 ligand motif-containing peptide to the SH3 domain-containing protein ARHGEF6 is found to be extremely low (9.897e-05), the lowest among all peptide-FHPI interactions evaluated in PepSite2 (**Fig H54 in S1 File**).

### OLPs found in GASP2 protein

GASP2_HUMAN (Q96D09) sequence contains the peptide ^444^EEEAIFGS**WFWDRDE**^458^ that has been shown to bind to human beta-1 adrenergic (ADRB1), muscarinic acetylcholine (ACM1) and calcitonin (CALCR) receptors [24]. The sequences of all interactors of the FHPIs- ADRB1_HUMAN (P08588), ACM1_HUMAN (P11229), and CALCR_HUMAN (P30988), were analyzed using MEME. In all the three cases, peptides from approximately the same region of GASP2 were reported, which when aligned were found to contain the OLP ^452^**WFWDRDE**ACFDLNPCPVY^469^ (**Fig G in S1 File**). Thus, we found that our method could provide a very near approximation of an actual experimentally validated motif instance that can bind to multiple proteins. The normalized OLPscore of this peptide was 1.47.

### Sequence matches observed by BLAST search

The results of BLAST homology search of the OLP sequences show several human proteins (neither of which was included in the SHPI networks used for MEME analysis) contain peptide sequences similar to the OLPs (**Table G in S1 File**). For example, the membrane-associated human protein OGFRL1 contains a peptide ^90^KRSFYAARD^98^ that is very similar to the MYC peptide ^371^KRSFFALRD^379^. These proteins may be investigated further for possible interactions with the FHPIs that have been predicted by our workflow to interact with the matching OLPs.

## Discussions

Identification of peptide regions in signaling hubs mediating disease-associated PPIs can be highly useful for edgetic perturbations on molecular regulatory networks using small-molecule inhibitors [44,45]. The smaller contact area seen in peptide-mediated PPIs, as compared to those mediated by large globular domains, offers a better possibility of targeting such interfaces with small chemical modulators for therapeutic intervention [5]. However, if the same peptide interface is involved in multiple PPIs, targeting one of the PPIs with orthosteric inhibitors may also affect other PPIs at the same site, leading to possible disruptions of essential PPI networks and pathways. Thus, it would be useful to predict the peptides capable of binding multiple interactors and study each of the PPIs separately, before targeting any one of these for therapeutic purposes. This would facilitate the design of safer and more specific PPI modulators in future.

In our proposed workflow, we have therefore taken a novel approach of aligning the peptides predicted to interact with different proteins to identify overlaps among them, so that these overlaps may represent sites interacting with multiple proteins. Hence, our framework deviates from the existing motif identification protocols, which only predict the linear peptide sequences capable of binding specific interactors, whereas we have tried to further predict whether these peptides might bind to multiple interactors. In this study, we have used MEME software to independently predict long peptide regions that may interact with each of the interactors of a CP, which were then aligned to reveal shorter peptides possibly interacting with multiple interactors. Here, we have also assumed that if a peptide is identified multiple times in the network-based approach, then the probability of that peptide to be involved in mediating PPIs will be higher. Thus, even though we have chosen a lenient E-value cut-off (<1.0) for the initial longer peptides, it is less likely that many of these longer peptide segments would share a shorter overlapping region completely by chance. We have used orthogonal methods to validate our findings. PepSite2 was used to structurally validate the peptide-mediated PPIs predicted by our method, whereas, GO and pathway enrichment analysis was done on the set of FHPIs predicted to interact with each OLP. However, the reliability of predictions from the PepSite2 server may have suffered due of the use of modeled structures in absence of experimentally determined 3D structures of the FHPI proteins. We have also searched for the presence of ELM-predicted motifs within the OLP sequences, and probed whether these OLPs fall in disordered and surface exposed regions of the corresponding proteins.

Few predicted high scoring OLPs from our study look promising for mutational studies affecting the underlying protein-protein interaction network and subsequent experimental validation. Two OLP sequences from C-terminal of MYC (^371^KRSFFALRD^379^ and ^392^KVVILKKATAY^402^) with high OLP scores have been predicted to bind with FBXW7. This protein was identified by Koch et al [46] in a large scale study of C-MYC interactions using tandem affinity purification. Interestingly, FBXW7 is a component of an E3 ubiquitin-protein ligase complex that mediates the ubiquitination and subsequent proteasomal degradation of target proteins. Since, the turnover rate of MYC is critical determinant of carcinogenesis [47], modulating these peptides may be useful in changing the half-life of c-MYC. A single stretch of MDM2 (456–475) contains two high scoring OLPs- ^456^GHLMACF^462^ and ^463^TCAKKLKKRNKPC^475^, both of which are predicted to bound to two important proteins: RNF8 and FKBP3. RNF8 plays significant roles in E2-E3 ubiquitin ligase complex and DNA damage response [48]. FKBP3, also known as FKBP25, contributes to regulation of the p53-MDM2 negative feedback loop [49]. Thus, there are strong motivations for experimental validation of these findings using AP-MS and peptide-protein interaction assays.

Nevertheless, there may be much more complexity associated with the prediction of peptide segments in cancer-associated hub proteins that mediate interactions with multiple partners. Although, we have considered significant motifs identified in multiple MEME runs, still there could be false positive interactors in the original PPI dataset. In our method, we have considered a minimal overlap region, but the actual interacting peptide segment can be longer or shorter in both N and C-terminal directions. Furthermore, while our method does provide information about the interacting partners, it does not investigate the binding domain or the structural parameters involved in the interactions. Nevertheless, the peptide-protein interactions predicted by our method may be thereafter studied in PepSite2 for predicting the probable binding site of the peptide on the interactor and the residues of the interacting surface that may be involved in such binding. In summary, we have made an attempt to develop a computational framework for identifying OLPs that can complement and accelerate the discovery of peptide regions mediating multiple interactions in date hub proteins.

## Supporting Information

S1 File **Table A:** Number of primary (FHPI) & secondary (SHPI) intra-species protein-protein interactions involving human MYC, APC and MDM2 as retrieved from the IntAct database.
**Table B:** List of significant motifs observed by de novo analysis in MYC.
**Table C:** List of significant motifs observed by de novo analysis in APC.
**Table D:** List of significant motifs observed by de novo analysis in MDM2.
**Table E:** Summary of FHPI protein 3D structures used for PepSite2 studies.
**Table F:** Results of docking of predicted OLPs against respective FHPI 3D structures in PepSite2.
**Table G:** Results of BLASTP search with the OLP sequences against all human proteins in the SwissProt database.
Fig A: Distribution of OLP scores for randomly generated FHPI networks.
Fig B: Degree distribution of primary interactors of MYC.
Fig C: Degree distribution of primary interactors of APC.
Fig D: Degree distribution of primary interactors of MDM2.
**Fig E: OLP identification in human APC protein.** (i) Three OLP sequences (each marked by a different background colour) were identified by Multiple Sequence Alignment of the peptide sequences in APC corresponding to all significant motifs (E-value<1.0) inferred by MEME from its SHPI network. (ii) Diagrammatic representation of an OLP sequence ^416^YCETCWEW^423^ (marked with blue background) identified in APC, which may interact with three FHPIs. The nodes representing oncoproteins have been marked with a red border.
**Fig F: OLP identification in human MDM2 protein.** (i) Six OLP sequences (each marked by a different background colour) were identified by Multiple Sequence Alignment of the peptide sequences in MDM2 corresponding to all significant motifs (E-value<1.0) inferred by MEME from its SHPI network. (ii) Diagrammatic representation of an OLP ^456^GHLMACF^462^ (marked with pink background) identified in MDM2, which may interact with four FHPIs. The nodes representing oncoproteins have been marked with a red border.
**Fig G: OLP identification in human GASP2 protein.** (i) OLP identified by Multiple Sequence Alignment of the peptide sequences in GASP2 corresponding to all significant motifs (E-value<1.0) inferred by MEME from the SHPI network from three FHPIs- ADRB1, ACM1 and CALCR. (ii) Diagrammatic representation of the OLP ^452^WFWDRDEACFDLNPCPVY^469^ that may be predicted to interact with the three FHPIs- ADRB1, ACM1 and CALCR.
Fig H: Screenshots of peptide-protein interactions predicted by PepSite2 server.
Images for peptides from MYC_HUMAN.Images for peptides from APC_HUMAN.Images for peptides from MDM2_HUMAN.
Fig I: Surface Accessibility predictions from SCRATCH prediction server for:
(i) MYC_HUMAN, (ii) APC_HUMAN, (iii) MDM2_HUMAN.
(DOC)
