# Supplementary material for: Computational Framework for Prediction of Peptide Sequences That May Mediate Multiple Protein Interactions in Cancer-Associated Hub Proteins
Source: PLoS One. 2016 May 24;11(5):e0155911. doi: 10.1371/journal.pone.0155911 (PMC4878775; doi:10.1371/journal.pone.0155911)
Supplement: S1 File — Table A: Number of primary (FHPI) & secondary (SHPI) intra-species protein-protein interactions involving human MYC, APC and MDM2 as retrieved from the IntAct database. Table B: List of significant motifs observed by de novo analysis in MYC. Table C: List of significant motifs observed by de novo analysis in APC. Table D: List of significant motifs observed by de novo analysis in MDM2. Table E: Summary of FHPI protein 3D structures used for PepSite2 studies. Table F: Results of docking of predicted OLPs against respective FHPI 3D structures in PepSite2. Table G: Results of BLASTP search with the OLP sequences against all human proteins in the SwissProt database. Fig A: Distribution of OLP scores for randomly generated FHPI networks. Fig B: Degree distribution of primary interactors of MYC. Fig C: Degree distribution of primary interactors of APC. Fig D: Degree distribution of primary interactors of MDM2. Fig E: OLP identification in human APC protein. (i) Three OLP sequences (each marked by a different background colour) were identified by Multiple Sequence Alignment of the peptide sequences in APC corresponding to all significant motifs (E-value<1.0) inferred by MEME from its SHPI network. (ii) Diagrammatic representation of an OLP sequence 416YCETCWEW423 (marked with blue background) identified in APC, which may interact with three FHPIs. The nodes representing oncoproteins have been marked with a red border. Fig F: OLP identification in human MDM2 protein. (i) Six OLP sequences (each marked by a different background colour) were identified by Multiple Sequence Alignment of the peptide sequences in MDM2 corresponding to all significant motifs (E-value<1.0) inferred by MEME from its SHPI network. (ii) Diagrammatic representation of an OLP 456GHLMACF462 (marked with pink background) identified in MDM2, which may interact with four FHPIs. The nodes representing oncoproteins have been marked with a red border. Fig G: OLP identification in human GASP2 protein. (i) OLP [file pone.0155911.s001.doc]

**Computational framework for identification of novel linear motifs prediction of peptide sequences that may mediate multiple protein interactions in cancer-associated hub proteins**

Debasree Sarkar, Piya Patra, Abhirupa Ghosh, Sudipto Saha

**Supporting Information:**

**List of Tables:**

**Table A:** Number of primary (FHPI) & secondary (SHPI) intra-species protein-protein interactions involving human MYC, APC and MDM2 as retrieved from the IntAct database.

**Table B:** List of significant motifs observed by de novo analysis in MYC.

**Table C:** List of significant motifs observed by de novo analysis in APC.

**Table D:** List of significant motifs observed by de novo analysis in MDM2.

**Table E:** Summary of FHPI protein 3D structures used for PepSite2 studies.

**Table F:** Results of docking of predicted OLPs against respective FHPI 3D structures in PepSite2.

**Table G:** Results of BLASTP search with the OLP sequences against all human proteins in the SwissProt database.

**List of Figures:**

**Fig A: Distribution of OLP scores for randomly generated FHPI networks.**

**Fig B: Degree distribution of primary interactors of MYC.**

**Fig C: Degree distribution of primary interactors of APC.**

**Fig D: Degree distribution of primary interactors of MDM2.**

**Fig E:** **OLP identification in human APC protein.** (i) Three OLP sequences (each marked by a different background colour) were identified by Multiple Sequence Alignment of the peptide sequences in APC corresponding to all significant motifs (E-value<1.0) inferred by MEME from its SHPI network. (ii) Diagrammatic representation of an OLP sequence ^416^YCETCWEW^423^ (marked with blue background) identified in APC, which may interact with three FHPIs. The nodes representing oncoproteins have been marked with a red border.

**Fig F: OLP identification in human MDM2 protein.** (i) Six OLP sequences (each marked by a different background colour) were identified by Multiple Sequence Alignment of the peptide sequences in MDM2 corresponding to all significant motifs (E-value<1.0) inferred by MEME from its SHPI network. (ii) Diagrammatic representation of an OLP ^456^GHLMACF^462^ (marked with pink background) identified in MDM2, which may interact with four FHPIs. The nodes representing oncoproteins have been marked with a red border.

**Fig G: OLP identification in human GASP2 protein.** (i) OLP identified by Multiple Sequence Alignment of the peptide sequences in GASP2 corresponding to all significant motifs (E-value<1.0) inferred by MEME from the SHPI network from three FHPIs- ADRB1, ACM1 and CALCR. (ii) Diagrammatic representation of the OLP ^452^WFWDRDEACFDLNPCPVY^469^ that may be predicted to interact with the three FHPIs- ADRB1, ACM1 and CALCR.

**Fig H: Screenshots of peptide-protein interactions predicted by PepSite2 server.**

(i) Images for peptides from MYC_HUMAN.

(ii) Images for peptides from APC_HUMAN.

(iii) Images for peptides from MDM2_HUMAN.

**Fig I: Surface Accessibility predictions from SCRATCH prediction server for:**(i) MYC_HUMAN, (ii) APC_HUMAN, (iii) MDM2_HUMAN.

**Table A:**

| **Name of the OP** | **Total no of Human FHPIs** | **Total no of Human SHPIs** |
| --- | --- | --- |
| MYC^1^ | 721 | 4849 |
| APC^2^ | 95 | 1000 |
| MDM2^3^ | 177 | 3047 |

^1^ As on 2-07-13, ^2^ As on 26-09-13, ^3^ As on 11-03-14.

**Table B:**

| **Motif seq in MYC_HUMAN** | **E-value** | **FHPI** | **Name of the SHPIs** | **Motifs observed in SHPIs** |
| --- | --- | --- | --- | --- |
| 233-GSPEPLVLHEETPPTTSSDS-252 | 1.20E-14 | SHANK2 | CRK, GRB2, NCK1, NLK, PIK3R1, PLCG1, SRC | GRB2, PLCG1, CRK, SRC, NCK1, PIK3R1, NLK |
| 371-KRSFFALRD-379 | 8.90E-11 | CNOT4 | EP300, UBE2D4, UBE2D2, UBE2E3, UBE2D3, UBE2N, UBE2W | UBE2D4, UBE2E3, UBE2D2, UBE2D3, UBE2W, EP300, UBE2N |
| 93-FSTADQLEMVTELLGGDMVNQSFICDPDDETFIKNIIIQDCMW-135 | 7.40E-13 | EXOC1 | SH3BP5, CCSER2, COLEC12, DISC1, DST, EXOC4, GOLGA4, IQCB1, KIAA1551, KIAA1731, MACF1, NUF2, SNAPIN, SNW1, SPTAN1, TRIM9, TRIO | MACF1, DST |
| 15-DYDSVQPYFYCDEEENFY -32 | 0.0059 | MSH3 | DBP, MSH2, SLX4, RCHY1 | DBP, MSH2, RCHY1 |
| 125-IKNIIIQDCMW-135 | 0.019 | RAB11FIP5 | ARRB1, ARRB2, GABARAPL2, SMAD3, TSC1 | GABARAPL2, ARRB1, SMAD3, ARRB2, TSC1 |
| 124-FIKNIIIQDCMWSGF-138 | 0.03 | BPTF | CBX6, H3F3A, HIST1H4A, SQSTM1 | HIST1H4A, CBX6, SQSTM1, H3F3A |
| 30-NFYQQQQQSELQPPAPSEDIWKKFEL-55 | 0.019 | KIF20B | YWHAB, CBX6, CBX8, RUVBL1 | CBX8, CBX6, RUVBL1 |
| 23-FYCDEEENFY-32 | 0.0025 | GIGYF2 | HSP90AA1, EIF4A2, MYC, RBM12, SNRPB, SNRPN, SNRPC, USP11, WBP11 | APC, SNRPC, USP11, HSP90AA1 |
| 112-NQSFICDPDDETFIKNIIIQDCMWSGFS-139 | 0.0004 | ILVBL | ATXN1, EGFR, ILK, PPP6R1, PPP6R2, TOM1, XRCC6 | PPP6R1, PPP6R2 |
| 112-NQSFICDPDDETFIKNIIIQDCMWSGFSA-140 | 0.00017 | PFDN5 | CALCOCO2, ETS1, HDAC1, IKBKE, ITSN1, MAP3K3, MAPK7, PPP2CB, PPP6R1, PPP6R2, PRPF4, GH1, TUBA3E, VHL | ITSN1, PPP6R2, PPP6R1 |
| 120-DDETFIKNIIIQDCM-134 | 0.018 | NFIL3 | AMOTL2, BHLHE40, CBX8, FHL1, NFIL3 | BHLHE40, NFIL3 |
| 32-YQQQQQSELQ-41 | 0.016 | KALRN | ATXN7, CACNA1A, COIL, DISC1, ENO3, GFI1B, NDEL1, FASLG | ATXN7, CACNA1A, DISC1 |
| 121-DETFIKNIIIQDCM-134 | 0.085 | IDH3B | PRMT1, ATXN2, IDH3A, MAPK6, PHLDA3, RAB35 | MAPK6, PRMT1, RAB35, IDH3A |
| 114-SFICDPDD-121 | 0.25 | NCAPG2 | NCAPH2, NEK6, PHLDA3, SMC2 | SMC2, PHLDA3, NEK6, NCAPH2 |
| 299-RCHVSTHQHNY-309 | 0.19 | NCAPG2 | NCAPH2, NEK6, PHLDA3, SMC2 | PHLDA3, NCAPH2, SMC2 |
| 115-FICDPDDETFIKNI-128 | 0.18 | EPC1 | ACTL6A, MRGBP, RUVBL1, RUVBL2, VPS82 | EPC1, ACTL6A, MRGBP |
| 128-IIIQDCMW-135 | 0.061 | MSH3 | DBP, MSH2, SLX4, RCHY1 | SLX4 |
| 109-DMVNQSFICD-118 | 0.11 | BPTF | CBX6, H3F3A, HIST1H4A, SQSTM1 | SQSTM1 |
| 8-TNRNYDLDYDSVQPYFYCDEEEN-30 | 0.12 | FASTKD2 | ICT1, PHLDA3, TSC22D1, TRAF6 | TRAF6 |
| 356-RRTHNVLERQRRNELKRSFFALRDQIPELENNEKAPKVVILKKATAY-402 | 0.0017 | FBXW7 | ARL6IP1, CCDC6, CCNE1, CUL1, EIF3E, FBXW7, HSP90AB1, KIAA1875, LINGO1, MMS22L, MYCN, SHPRH, SKP1, SUMF2, NFKB2 | MYCN |
| 133-CMWSGFSAAAK-143 | 0.17 | HIGD1A | EPB41, CASP4, PAK7, SNRPB, TSC22D1, USP32 | CASP4, USP32 |
| 10-RNYDLDYD-17 | 0.32 | IL4R | ARIH2, IL13RA1, IL2RG, IL4R, IL4, PTPN6, INPP5D, STAT6 | IL13RA1, IL2RG, IL4R, PTPN6 |
| 129-IIQDCMWSGFSA-140 | 0.016 | FBXW7 | ARL6IP1, CCDC6, CCNE1, CUL1, EIF3E, FBXW7, HSP90AB1, KIAA1875, LINGO1, MMS22L, MYCN, SHPRH, SKP1, SUMF2, NFKB2 | MYCN |
| 109-DMVNQSFICDPDDETFIKNIIIQDCMWSG-137 | 0.2 | MRPL14 | COX15, MCC, ICT1, IKBKE, TNIK, VHL | IKBKE |
| 392-KVVILKKATAYI-403 | 0.3 | TCF12 | BMF, HEXIM2, LAMTOR5, MYF6, PRSS23, TAL1, TRIM33 | MYF6, TAL1 |
| 128-IIIQDCMW-135 | 0.16 | NUP188 | G3BP1, HIPK4, ICK, IQCB1, CHM, SLX4, PTP4A3, VCAM1 | SLX4 |
| 127-NIIIQDCMW-135 | 0.41 | ZCCHC11 | CBX1, GABARAPL2, LIN28B, LIN28A | GABARAPL2 |
| 128-IIIQDCMW-135 | 0.27 | KPNA4 | ARRB2, CBX1, CBX3, CBX5, CSNK2A1, HDAC1, MED26, RAC1, RECQL, SLX4 | SLX4 |
| 109-DMVNQSFICD-118 | 0.55 | HCFC2 | PPP2R2B, ASH2L, ESR2, FOXO3, IRF2, KMT2A, MEN1, RBBP5, SIRT1, UTP18 | FOXO3, ASH2L, UTP18 |
| 22-YFYCDEEENFY-32 | 0.58 | NFIL3 | AMOTL2, BHLHE40, CBX8, FHL1, NFIL3 | FHL1 |
| 299-RCHVSTHQHNY-309 | 0.53 | MYO1B | BRF2, CIAO1, E2F3, ESR1, GRB2, EIF4A2, MAP3K3, MAP1LC3A, RIPK3, TSC22D1, PTP4A3, ATP6V1A | MAP3K3, TSC22D1 |
| 130-IQDCMWSG-137 | 0.69 | SHANK2 | CRK, GRB2, NCK1, NLK, PIK3R1, PLCG1, SRC | NLK, SRC |
| 7-FTNRNYDLDYDSVQPYFYCDEEENFY-32 | 0.77 | TCF12 | BMF, HEXIM2, LAMTOR5, MYF6, PRSS23, TAL1, TRIM33 | PRSS23 |
| 117-CDPDDETFIKNIIIQDC-133 | 0.96 | RNF130 | ARPC4, CDC5L, FGFR3, SCN2A | SCN2A |

**Table C:**

| **Motif sequence in APC_HUMAN** | **E-value** | **FHPI** | **Name of the SHPIs** | **Motifs observed in SHPIs** |
| --- | --- | --- | --- | --- |
| 422-EWQEAH-427 | 0.0037 | NCKAP5 | ABL1, FYN, GRB2, NCK1, VAV2 | FYN, NCK1, VAV2, GRB2, ABL1 |
| 2097-ENFDWK-2102 | 0.011 | RBM4B | GABARAPL2, POLR2M, HNRNPM, MRPS9 | HNRNPM, MRPS9, GABARAPL2, POLR2M |
| 155-KDWYYA-160 | 0.029 | CYTH2 | ADORA2A, ARF1, CCDC120, EGFR, GRB2, ATP6V0A2 | CCDC120, ARF1, EGFR, ATP6V0A2, GRB2, ADORA2A |
| 415-AYCETCWEWQ-424 | 0.0025 | GIGYF2 | HSP90AA1, EIF4A2, MYC, RBM12, SNRPB, SNRPN, SNRPC, USP11, WBP11 | SNRPC, USP11, MYC, HSP90AA1 |
| 416-YCETCWEW-423 | 0.11 | EPAS1 | ARNT, BBS1, BBS2, BBS4, ARNTL2, ARNTL, EIF3E, EP300, EWSR1, PKM, MAX, MEF2C, PIAS2, STAT5A, SUMO1, VHL | STAT5A, EP300 |
| 416-YCETCWEWQEAH-427 | 0.74 | ANKRD17 | ADH6, BAP1, NFKBIE, MDM2, SLX4, VCAM1 | MDM2 |
| 155-KDWYYAQ-161 | 0.95 | GIGYF2 | HSP90AA1, EIF4A2, MYC, RBM12, SNRPB, SNRPN, SNRPC, USP11, WBP11 | USP11, SNRPC |

**Table D:**

| **Motif sequence in MDM2_HUMAN** | **E-value** | **FHPI** | **Name of the SHPIs** | **Motifs observed in SHPIs** |
| --- | --- | --- | --- | --- |
| 475-CPVCRQPI-482 | 3.4E-06 | PHF7 | ARIH2, DTX3L, IKBKG, RNF111, RNF14, RNF34, SRPK1, TRIM26, TRIM41 | RNF111, RNF14, RNF34, ARIH2, TRIM26, TRIM41, DTX3L, IKBKG, SRPK1 |
| 456-GHLMACFTCAKKLKKRNKPCPVC-478 | 3.7E-08 | RNF8 | ATM, BRCA1, CCDC85B, CEP44, CHD4, EXOSC2, H2AFX, ICP0, MCPH1, MDC1, NBN, ORC2, PNMA2, RNF8, TP53BP1, UBE2D2, UBE2N, UBE2W, WASL | RNF8, ICP0, BRCA1, CHD4, ATM |
| 303-WKCTSCNEMN-312 | 0.0013 | HRSP12 | MT-CO2, DEAF1, HPD, SERPINA5, SPEN, TMEM176A, TRAF6, FTSJ1, HIVEP1 | HIVEP1, DEAF1, TRAF6, MT-CO2, TMEM176A |
| 463-TCAKKLKKRNKPCPVCRQPIQ-483 | 0.05 | HLA-DMB | HLA-DRB1, CUL3, HLA-DMA, TEAD1 | HLA-DRB1, TEAD1 |
| 435-IEPCVICQ-442 | 0.11 | PHF7 | ARIH2, DTX3L, IKBKG, RNF111, RNF14, RNF34, SRPK1, TRIM26, TRIM41 | DTX3L, TRIM41, RNF34, RNF111, TRIM26, ARIH2, RNF14, SRPK1 |
| 449-CIVHGKTGHLMACF-462 | 0.028 | GLTSCR2 | EIF2AK2, HAP1, PPP1CA, SLX4, USP45, UIMC1 | USP45, EIF2AK2 |
| 300-ADYWKCTSCNEMNPPLPSHCNRCWALRENWLP-331 | 0.0034 | MAP4K4 | GBP3, HIST2H2BE, HSP90AB1, KIF26B, KIF5A, PRKCE, MOB1B, NME7, PPP1CA, PPP1CC, PPARG, SLC9A1, ZRANB1 | ZRANB1 |
| 311-MNPPLPSHCNRC-322 | 0.24 | PIM1 | BMX, SHMT1, HSP90AB1, RPS19, STAT3 | STAT3, RPS19 |
| 304-KCTSCNEMNPPLPSHCNRCW-323 | 0.073 | PIM2 | ATXN1, FBXO18, GSK3B, HNRNPH3, HSP90AB1, NDUFB8, IKBKG, RPL21 | NDUFB8 |
| 438-CVICQGRPKNGCIVHGKTGHLMACFTC-464 | 0.17 | TSNAX | CKAP4, DMWD, EP300, GADD45G, IKBKE, LIN37, MRFAP1L1, PBXIP1, RALYL, TSNAX, TSN | EP300, GADD45G |
| 438-CVICQGRPKNGCIVHGKTGHLMACFTCAKKLKKRNKPC-475 | 0.51 | FKBP3 | HLA-B, MCC, PARP2, SRPK1, TNIK | SRPK1, TNIK |
| 301-DYWKCTSCNEMNPPLPSHCNRCWALRENW-329 | 0.12 | ARHGEF6 | ADAM15, ARRB2, GPRASP2, GIT1, MCM7, NAA10, PAK1, PAK2, SH2D1A, SH3GL3, TMEM108, ZNF580 | NAA10 |
| 460-ACFTCAKKLKKRNKPCPVCR-479 | 0.42 | JUND | ACTR3, SLC25A12, ETS1, FOSL1, FOS, JDP2, RFWD2, SEPT2 | RFWD2, SLC25A12 |
| 308-CNEMNPPLPSHCNRCWA-324 | 0.37 | YY1AP1 | PRKAB2, ATXN1, EWSR1, MAPK8IP2, MAPK14, ZFAT | EWSR1 |
| 305-CTSCNEMNPPLPSHCNRCW-323 | 0.46 | NEFM | ETS1, FOS, IQCB1, JUN, NR4A2, PTBP3, RAC3, RB1, SMAD2, SQSTM1, TNIK | SQSTM1, SMAD2 |
| 302-YWKCTSCNEMNPPLPSHCNRCWALRENWL-330 | 0.52 | ZNF326 | AGO1, BRINP1, HDAC11, MAP1LC3B, MAP1LC3A, HNRNPA1, POLR2A, SNW1 | BRINP1 |
| 302-YWKCTSCNEMNPPLPSHC-319 | 0.65 | TSNAX | CKAP4, DMWD, EP300, GADD45G, IKBKE, LIN37, MRFAP1L1, PBXIP1, RALYL, TSNAX, TSN | EP300 |
| 308-CNEMNPPLPSHCNRCW-323 | 0.84 | USP2 | BEGAIN, BEND5, CCDC85B, HOOK2, KRT15, LONP1, RRP15, TRAF2, UBC | TRAF2 |

**Table E:**

| **Name of the CP** | **Name of the FHPI** | **PDB ID** | **Model Source** | **Sequence identity (%)** | **Number of amino acid residues in the FHPI** | **Residues covered by the structure** |
| --- | --- | --- | --- | --- | --- | --- |
| MYC | CNOT4 | 1E4U.A | - | - | 575 | 1- 78 |
|  | FBXW7 | 2OVR.B | - | - | 707 | 263-707 |
|  | EXOC1 | 3A58.A(template) | ModBase | 20 | 894 | 28-152 |
|  | ILVBL | 2UZ1.A(template) | ModBase | 31 | 632 | 54-620 |
|  | PFDN5 | 1FXK.C(template) | ModBase | 28 | 154 | 7-150 |
|  | NCAPG2 | 4HMY.B | Reliable model not found | - | 1,143 | - |
|  | MRPL14 | 3J7Y.L | - | - | 145 | 1-145 |
|  | RAB11FIP5 | 4D0G.C(template) | SwissModel | 51 | 653 | 604-648 |
|  | BPTF | 2RI7.A | - | - | 3,046 | 2865-3033 |
|  | MSH3 | 3THX.B | - | - | 1,137 | 219-1134 |
|  | NUP188 | 4KF7.A(template) | ModBase | 16 | 1,749 | 60-938 |
|  | ZCCHC11 | 2IHX.A(template) | ModBase | 48 | 1,644 | 1295-1372 |
|  | KPNA4 | 4UAE.A | - | - | 521 | 68-487 |
|  | KIF20B | 1F9W.B(template) | SwissModel | 44 | 1,820 | 376-474 |
|  | KALRN | 2KR9.A(template) | SwissModel | 99 | 2,985 | 1279-1459 |
|  | TCF12 | 2QL2.C(template) | SwissModel | 87 | 682 | 578-637 |
|  | GIGYF2 | 4KJZ.A(template) | ModBase | 49 | 1,299 | 719-778 |
|  | FASTKD2 | 1Z3H.B | Reliable model not found | - | 710 | - |
|  | NFIL3 | 1DH3.A(template) | ModBase | 43 | 462 | 78-126 |
|  | IL4R | 1IAR.B | - | - | 825 | 26-232 |
|  | MYO1B | 4L79.A(template) | SwissModel | 97 | 1,136 | 5-728 |
| APC | GIGYF2 | 4KJZ.A(template) | ModBase | 49 | 1,299 | 719-778 |
|  | EPAS1 | 3F1P.A | - | - | 870 | 239-350 |
|  | ANKRD17 | 1N0R.A(template) | ModBase | 50 | 2,603 | 303-425 |
|  | NCKAP5 | 1DG3.A(template) | ModBase | 32 | 1,909 | 36-259 |
|  | CYTH2 | 1R8S.E | - | - | 400 | 50-252 |
| MDM2 | RNF8 | 2PIE.A | - | - | 485 | 13-146 |
|  | GLTSCR2 | 4MH6.A | Reliable model not found | - | 478 | - |
|  | TSNAX | 3PJA.J | - | - | 290 | 1-290 |
|  | FKBP3 | 1PBK.A | - | - | 224 | 109-224 |
|  | HLA-DMB | 2BC4.B | - | - | 263 | 19-256 |
|  | JUND | 1JNM.A(template) | ModBase | 88 | 347 | 270-325 |
|  | PHF7 | 1WEQ.A(template) | ModBase | 82 | 381 | 228-307 |
|  | HRSP12 | 1ONI.A | - | - | 137 | 2-137 |
|  | MAP4K4 | 4OBO.A | - | - | 1,239 | 2-328 |
|  | PIM2 | 4X7Q.A | - | - | 311 | 1-311 |
|  | ARHGEF6 | 1UJY.A | - | - | 776 | 160-222 |
|  | NEFM | 1GK4.A(template) | SwissModel | 68 | 916 | 330-407 |
|  | ZNF326 | 1NAY.A | Reliable model not found | - | 582 | - |
|  | PIM1 | 3A99.A | - | - | 404 | 106-404 |
|  | YY1AP1 | 1OQY.A | Reliable model not found | - | 796 | - |
|  | USP2 | 3NHE.A | - | - | 605 | 258-605 |

**Table F:**

| **Name of the CP** | **OLP sequence** | **Name of the FHPIs** | **Residues of FHPI involved in binding** | **p value** | **Inferred Significance*** |
| --- | --- | --- | --- | --- | --- |
| MYC | 371-KRSFFALRD-379 | CNOT4 | E19, F40, H43, T47 | 0.1576 | Moderately Significant |
|  |  | FBXW7 | Y2291, F2295 | 0.09191 | Moderately Significant |
|  | 114-SFICDPDD-121 | EXOC1 | C36, F37, L38, C39, V52, K54, F63, L92, W100, F111, I115 | 0.2623 | Not significant |
|  |  | ILVBL | F387, w388 | 0.2637 | Not significant |
|  |  | PFDN5 | G89, y90, y91 | 0.4178 | Not significant |
|  |  | NCAPG2 | -- | Model not built | -- |
|  |  | MRPL14 | Y51, h52, k78, g79 | 0.3578 | Not significant |
|  | 128-IIIQDCMW-135 | EXOC1 | F37, l38, c39, f63, f90, l92, w100, f111 | 0.1183 | Moderately Significant |
|  |  | RAB11FIP5 | H622, l626, y629 | 0.2192 | Not significant |
|  |  | BPTF | Y10, e17, y23, w32 | 0.03816 | Highly Significant |
|  |  | ILVBL | F387, w388 | 0.1056 | Moderately Significant |
|  |  | PFDN5 | Y43, q118 | 0.05528 | Significant |
|  |  | MSH3 | F1098, w1102 | 0.02065 | Highly Significant |
|  |  | MRPL14 | K34, h59, y61, l74, k83, d110 | 0.1401 | Moderately Significant |
|  |  | NUP188 | A126, Y129, Y130, R133, E179, T187, W188, M195, Q199, W203, Q206 | 0.001193 | Highly Significant |
|  |  | ZCCHC11 | R1311, k1312, e1328, f1360 | 0.448 | Not significant |
|  |  | KPNA4 | R96, w137, w179, n183, n219, w222 | 0.002434 | Highly Significant |
|  | 32-YQQQQQSELQ-41 | KIF20B | Y460, y463 | 0.05348 | Significant |
|  |  | KALRN | W1309, y1337, h1401 | 0.03485 | Highly Significant |
|  | 392-KVVILKKATAY-402 | FBXW7 | Y2291, f2295 | 0.3029 | Not significant |
|  |  | TCF12 | L617, l620, h621 | 0.8503 | Not significant |
|  | 23-FYCDEEEN-30 | MSH3 | F1098, w1102 | 0.06885 | Significant |
|  |  | GIGYF2 | R747, r755, e759 | 0.9977 | Not significant |
|  |  | FASTKD2 | -- | Model not built | -- |
|  |  | NFIL3 | F126 | 0.5086 | Not significant |
|  |  | TCF12 | R578, r579 | 0.9332 | Not significant |
|  | 10-RNYDLDYD-17 | FASTKD2 | -- | Model not built | -- |
|  |  | IL4R | Y13, f41, S80, y127, y183 | 0.1391 | Moderately Significant |
|  |  | TCF12 | F597, l617, l620, h621 | 0.9304 | Not significant |
|  | 299-RCHVSTHQHNY-309 | NCAPG2 | -- | Model not built | -- |
|  |  | MYO1B | Y715, w718 | 0.1681 | Moderately Significant |
| APC | 416-YCETCWEW-423 | GIGYF2 | R747, e751, r755 | 0.9374 | Not significant |
|  |  | EPAS1 | S246, h248, d251, m252, f254, s276, a277, y278, y281, m289, t290, h293, l296, y307, m309, t321, i337, c339, n341 | 0.1145 | Moderately Significant |
|  |  | ANKRD17 | H396, f400, y410 | 0.5264 | Not significant |
|  | 422-EWQEAH-427 | NCKAP5 | K239, f243, d244 | 0.4864 | Not significant |
|  |  | ANKRD17 | H396, f400 | 0.2433 | Not significant |
|  | 155-KDWYYA-160 | CYTH2 | W149, e241, k244 | 0.3792 | Not significant |
|  |  | GIGYF2 | R747, r750 | 0.993 | Not significant |
| MDM2 | 456-GHLMACF-462 | RNF8 | M12, l31, w129, y133 | 0.01761 | Highly Significant |
|  |  | GLTSCR2 | -- | Model not built | -- |
|  |  | TSNAX | F39, f42, l46 | 0.1709 | Moderately Significant |
|  |  | FKBP3 | C133, W134, Y135, D146, N148, P161, L162, K170, V171, I172, W175, L179, y198, f216, v218 | 0.01931 | Highly Significant |
|  | 463-TCAKKLKKRNKPC-475 | RNF8 | M12, g15, r16, s17, l31, w129, y133 | 0.07304 | Significant |
|  |  | HLA-DMB | F86, l90, y123, w154, y156 | 0.01368 | Highly Significant |
|  |  | FKBP3 | C133, w134, y135, f145, d146, k154, l162, w175, l179, l190, y198, i208, l214, f216, e217, v218, e219 | 0.1351 | Moderately Significant |
|  |  | JUND | K287, r288, e291, r295 | 0.9997 | Not significant |
|  | 475-CPVCR-478 | PHF7 | W231 | 0.04128 | Highly Significant |
|  |  | HLA-DMB | T83, f86, w87, l90, w154 | 0.002625 | Highly Significant |
|  |  | JUND | R279, r286 | 1.0 | Not significant |
|  | 305-CTSCN-309 | HRSP12 | T30, y32 | 0.3474 | Not significant |
|  |  | MAP4K4 | F25, q49, t84, y85, y86, g87, v104 | 0.2628 | Not significant |
|  |  | PIM2 | W88, w105 | 0.06501 | Significant |
|  |  | ARHGEF6 | T22, e26, w45, w56 | 0.005455 | Highly Significant |
|  |  | NEFM | R371, w375 | 0.2871 | Not significant |
|  |  | ZNF326 | -- | Model not built | -- |
|  |  | TSNAX | F39, f42 | 0.06092 | Significant |
|  | 311-MNPPLPSHC-319 | MAP4K4 | F25, h44, q49, t84, t85, g87, v104 | 0.07288 | Significant |
|  |  | PIM1 | H265, w269, e283, h287 | 0.03425 | Highly Significant |
|  |  | PIM2 | V84, w88, w105, f106 | 0.007897 | Highly Significant |
|  |  | ARHGEF6 | F17, f19, d25, e26, w45, w56, p58, y61 | 9.897e-05 | Highly Significant |
|  |  | YY1AP1 | -- | Model not built | -- |
|  |  | NEFM | R371, w375 | 0.4385 | Not significant |
|  |  | ZNF326 | -- | Model not built | -- |
|  |  | TSNAX | F39, f42 | 0.1849 | Moderately Significant |
|  |  | USP2 | D267, h370, w400, y403, l404, e407, d408, s409, g412, v416, g417, p437, w439 | 0.1075 | Moderately Significant |
|  | 438-CVICQ-442 | PHF7 | W231 | 0.1627 | Moderately Significant |
|  |  | TSNAX | F39, f42, l46 | 0.02254 | Highly Significant |
|  |  | FKBP3 | C133, w134, y135, d146, l162, f164, i172, w175, l179, y198, f216, v218 | 0.01598 | Highly Significant |

* Highly Significant (p-values<0.05); Significant (0.05<p-values<0.125); Moderately Significant (0.125<p-values<0.175); Not significant (p-values>0.175)

**Table G**:

| **Name of the CP** | **OLP sequence in CP** | **Name of the FHPIs that may interact with the OLP** | **Top hit from BLAST search of OLP sequence in human protein sequences from SwissProt database** | | **Query Coverage** | **Sequence Identity** |
| --- | --- | --- | --- | --- | --- | --- |
|  |  |  | **Sequence** | **Name of the protein** |  |  |
| MYC | 371-KRSFFALRD-379 | CNOT4, FBXW7 | 90-KRSFYAARD-98 | OGFRL1 | 100% | 78% |
|  | 114-SFICDPDD-121 | EXOC1, ILVBL, PFDN5, NCAPG2, MRPL14 | 51-FICDPD-56 | GJE1 | 75% | 100% |
|  | 128-IIIQDCMW-135 | EXOC1, RAB11FIP5, BPTF, ILVBL, PFDN5, MSH3, MRPL14, NUP188, ZCCHC11, KPNA4 | 162-IIEDCM-167 | BAG5 | 75% | 83% |
|  | 32-YQQQQQSELQ-41 | KIF20B, KALRN | 739-QQQQQSPLQ-747 | NFAT5 | 90% | 89% |
|  | 392-KVVILKKATAY-402 | FBXW7, TCF12 | 257-ILKMATAY-264 | BCO1 | 72% | 88% |
|  | 23-FYCDEEEN-30 | MSH3, GIGYF2, FASTKD2, NFIL3, TCF12 | 307-CNEEEN-312 | AGGF1 | 75% | 83% |
|  | 10-RNYDLDYD-17 | FASTKD2, IL4R, TCF12 | 550-RKYNLDY-556 | ZNF804B | 87% | 71% |
|  | 299-RCHVSTHQHNY-309 | NCAPG2, MYO1B | 329-CHISSHQHD-337 | F8 | 81% | 67% |
| APC | 416-YCETCWEW-423 | GIGYF2, EPAS1, ANKRD17 | 1228-ETALACWEW-1236 | PI4KA | 75% | 67% |
|  | 422-EWQEAH-427 | NCKAP5, ANKRD17 | 45-DWQEAH-50 | PATL1 | 100% | 83% |
|  | 155-KDWYYA-160 | CYTH2, GIGYF2 | 133-DWYYA-137 | FYCO1 | 83% | 100% |
| MDM2 | 456-GHLMACF-462 | RNF8, GLTSCR2, TSNAX, FKBP3 | 86-GHLMCAGCF-94 | CYHR1 | 100% | 67% |
|  | 463-TCAKKLKKRNKPC-475 | RNF8, HLA-DMB, FKBP3, JUND | 201-TCAEKLKKR-209 | MAP2K7 | 69% | 89% |
|  | 475-CPVCR-478 | PHF7, HLA-DMB, JUND | 78-CPVCR-82 | BRI3 | 100% | 100% |
|  | 305-CTSCN-309 | HRSP12, MAP4K4, PIM2, ARHGEF6, NEFM, ZNF326, TSNAX | 43-CTSCN-47 | CGGBP1 | 100% | 100% |
|  | 311-MNPPLPSHC-319 | MAP4K4, PIM1, PIM2, ARHGEF6, YY1AP1, NEFM, ZNF326, TSNAX, USP2 | 989-PPLPSH-994 | GRM1 | 66% | 100% |
|  | 438-CVICQ-442 | PHF7, TSNAX, FKBP3 | 380-CVICQ-384 | RNF26 | 100% | 100% |

**
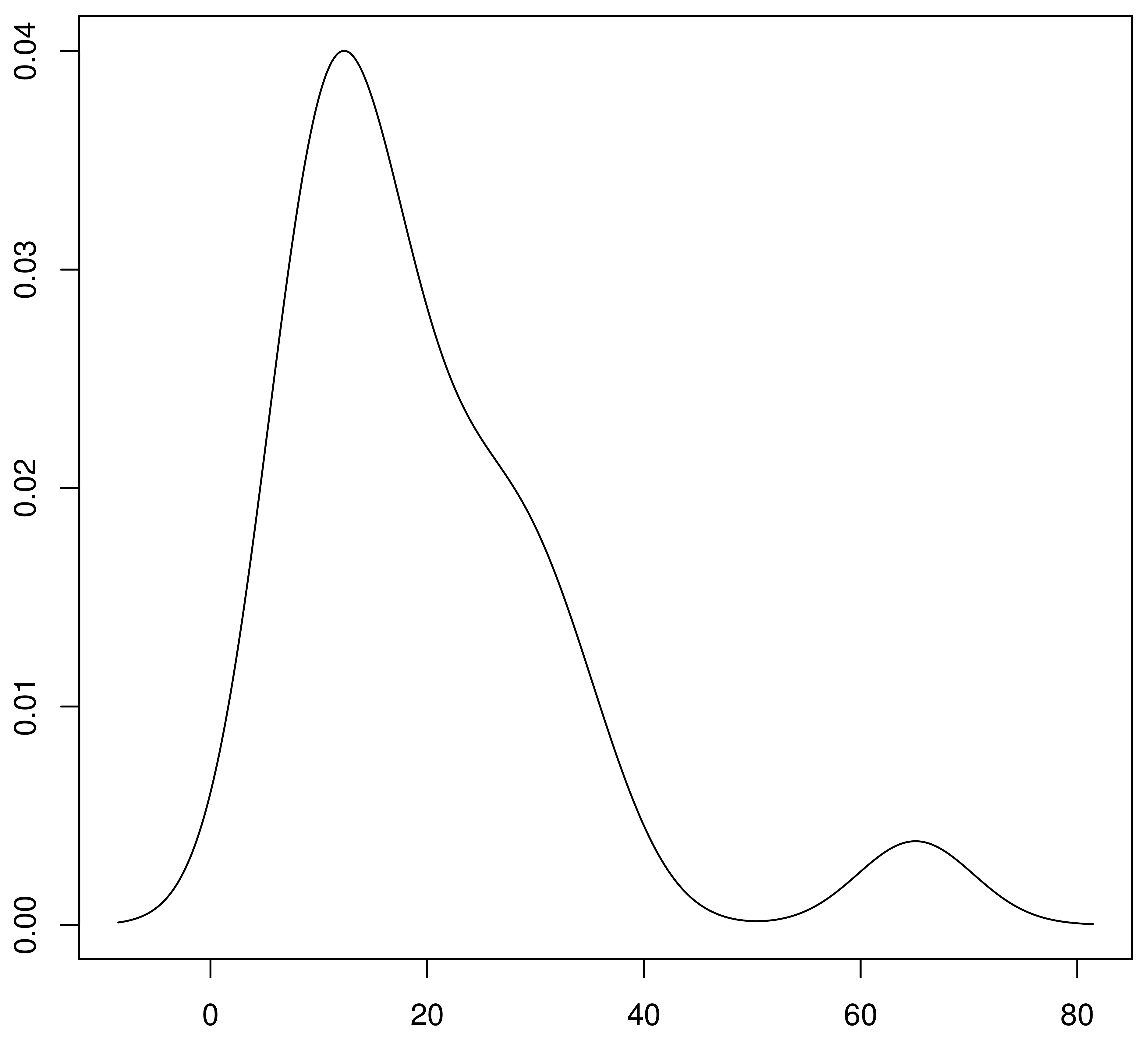

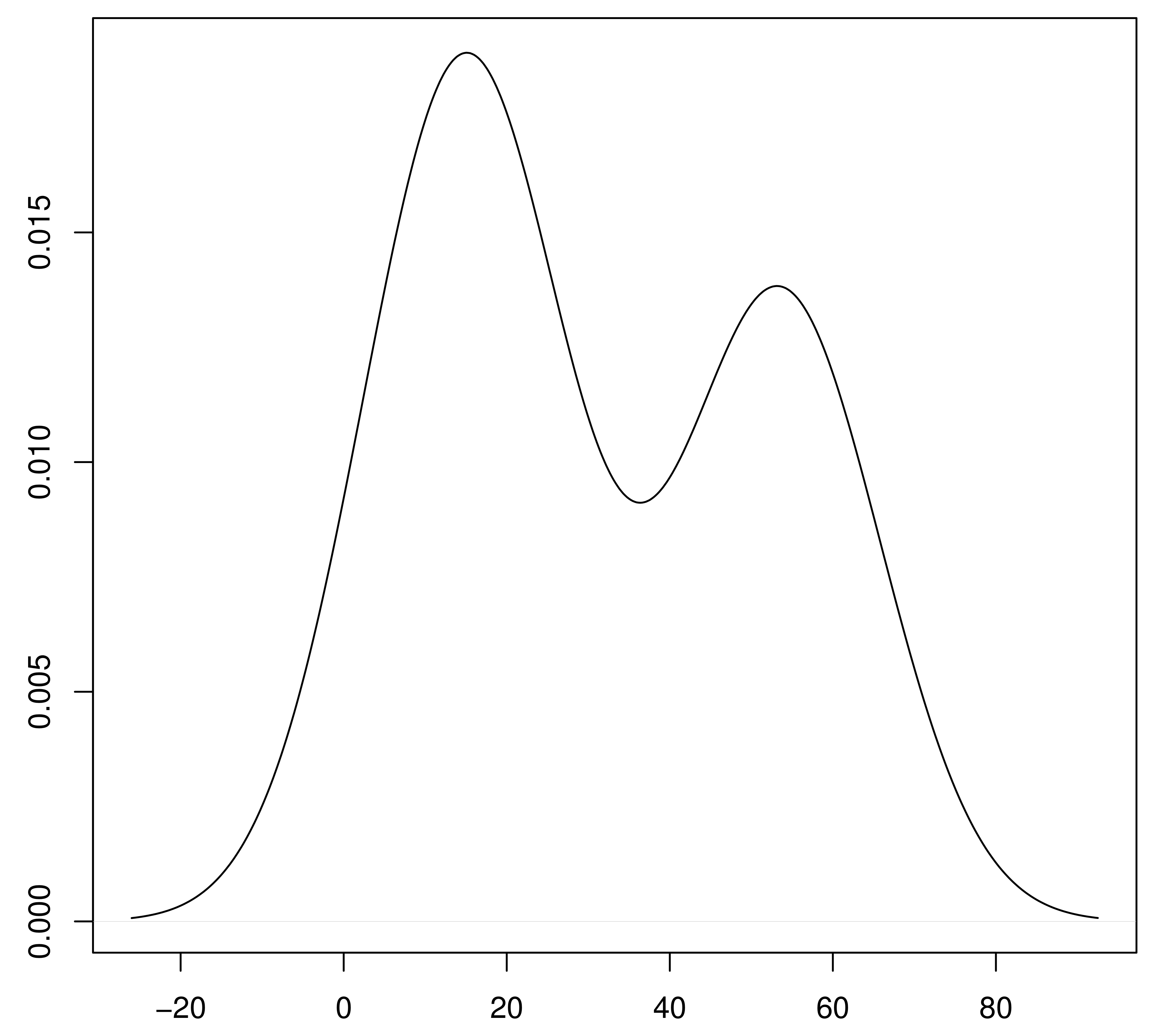
**

(i) (ii)


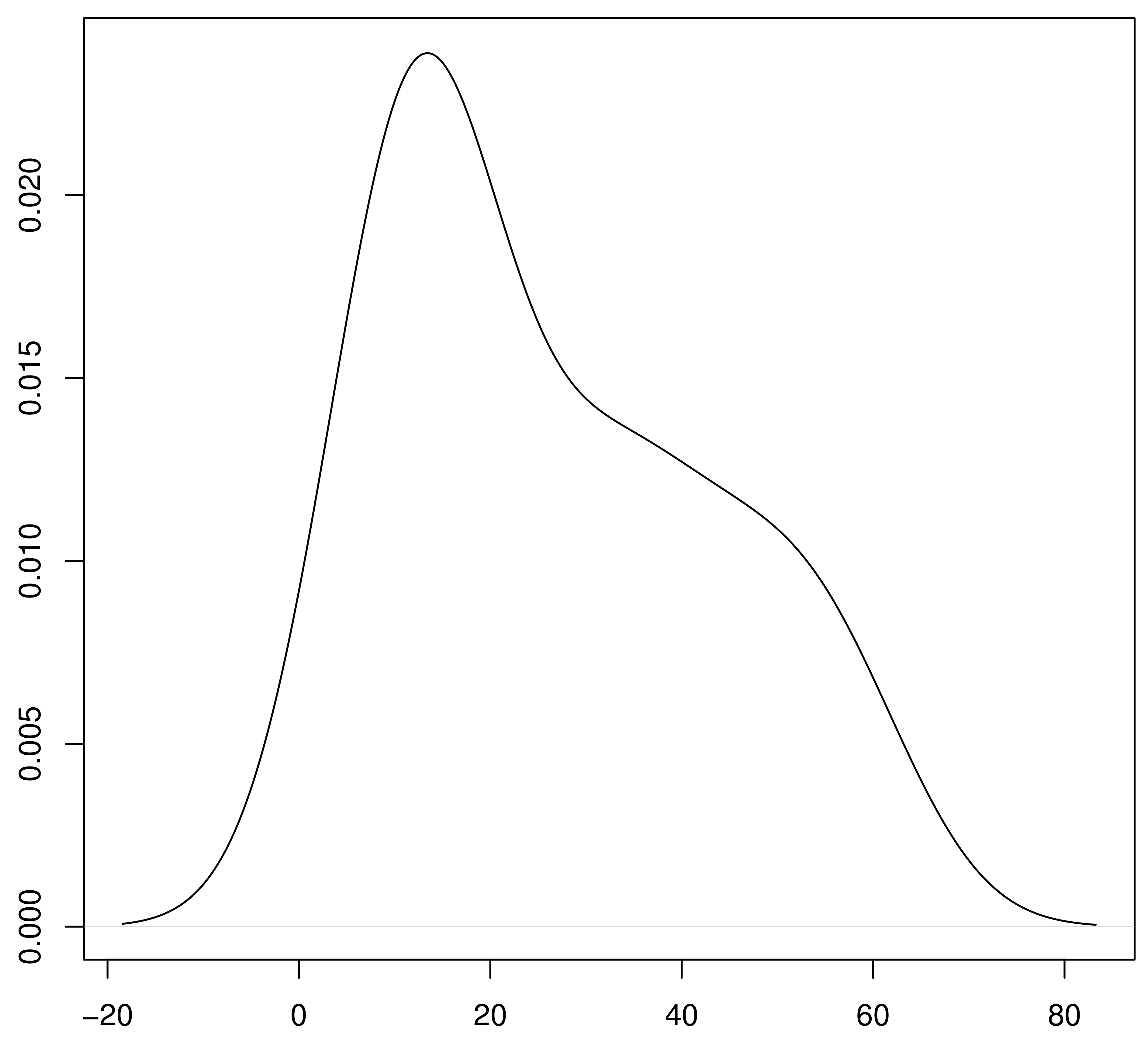

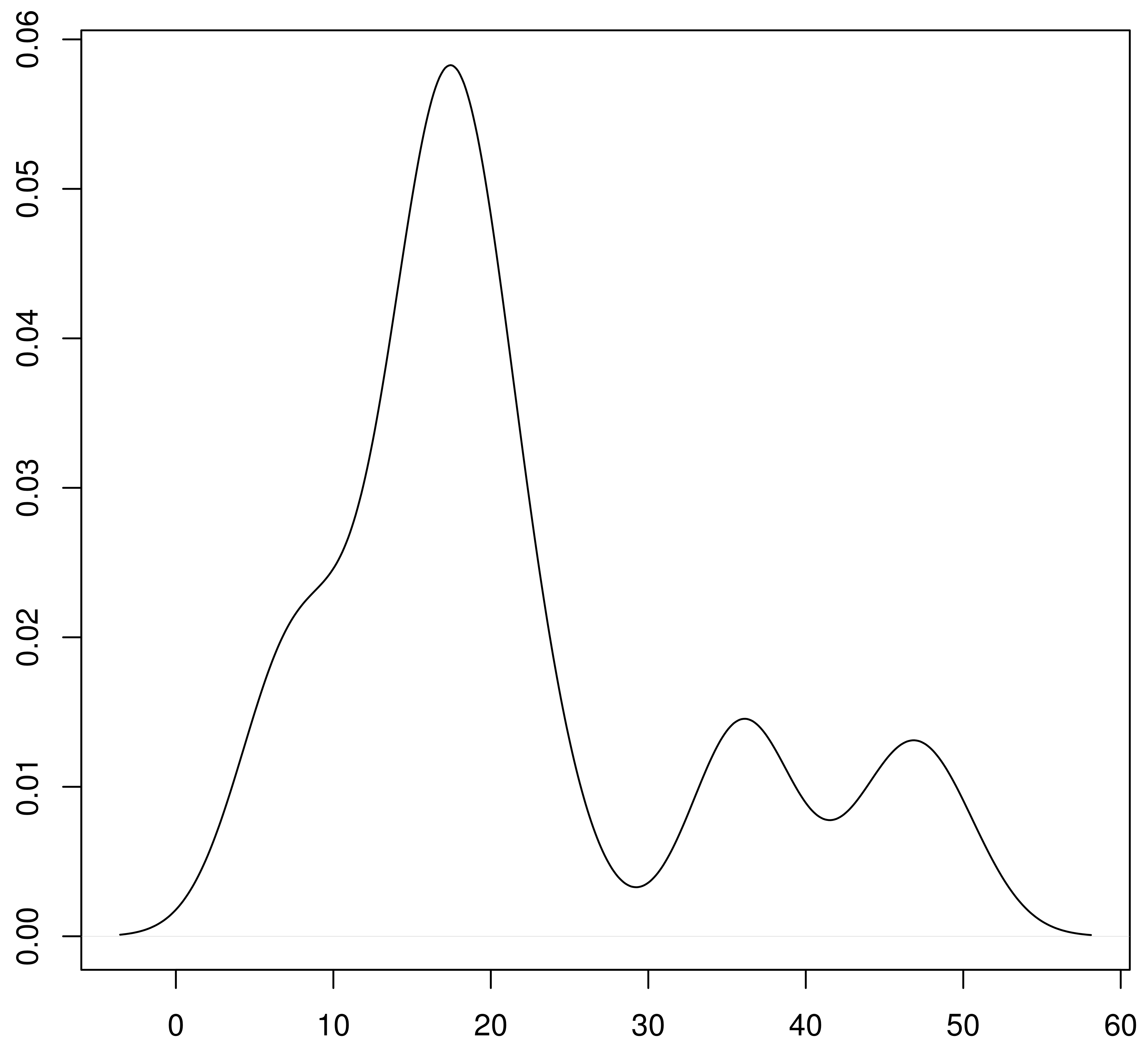


(iii) (iv)

**Fig A**

**
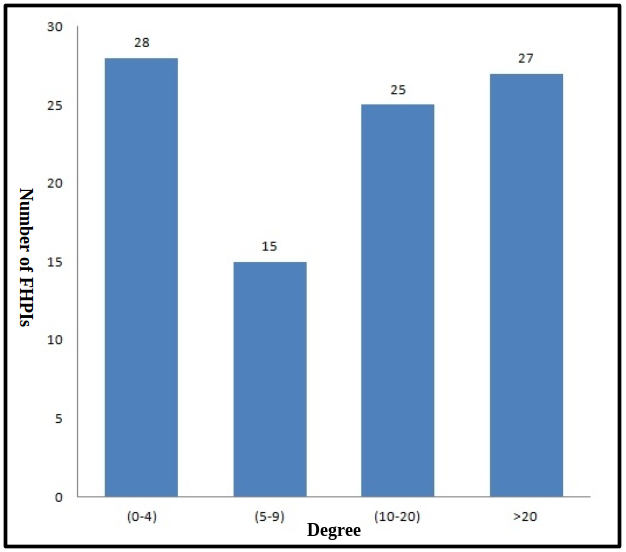

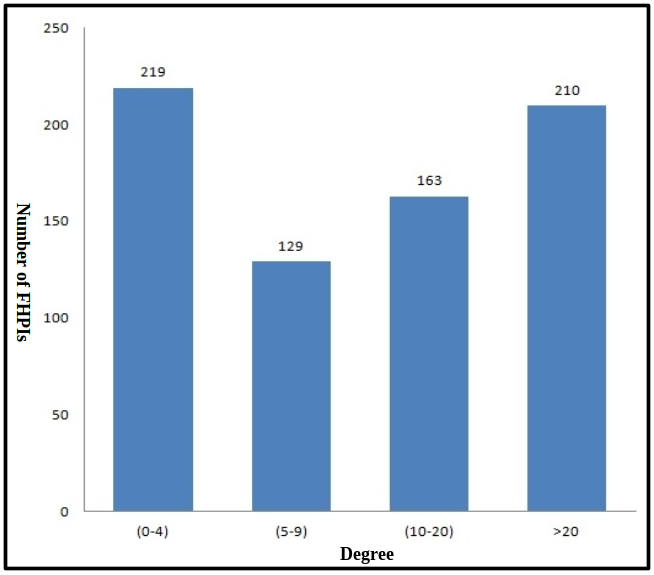
**

**Fig B Fig C**

**
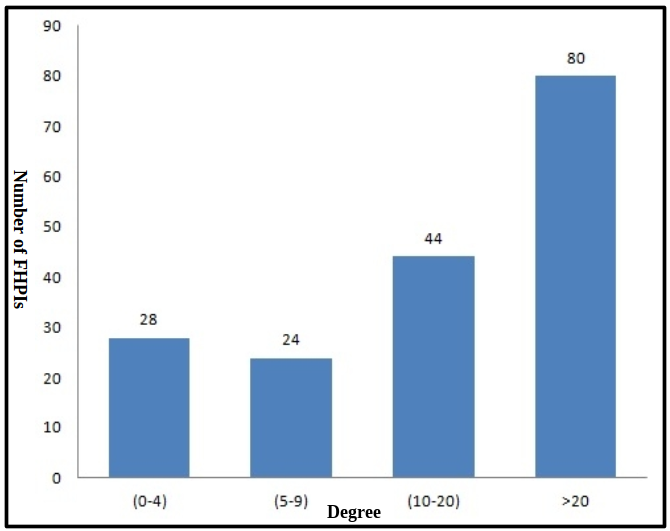
**

**Fig D**

**
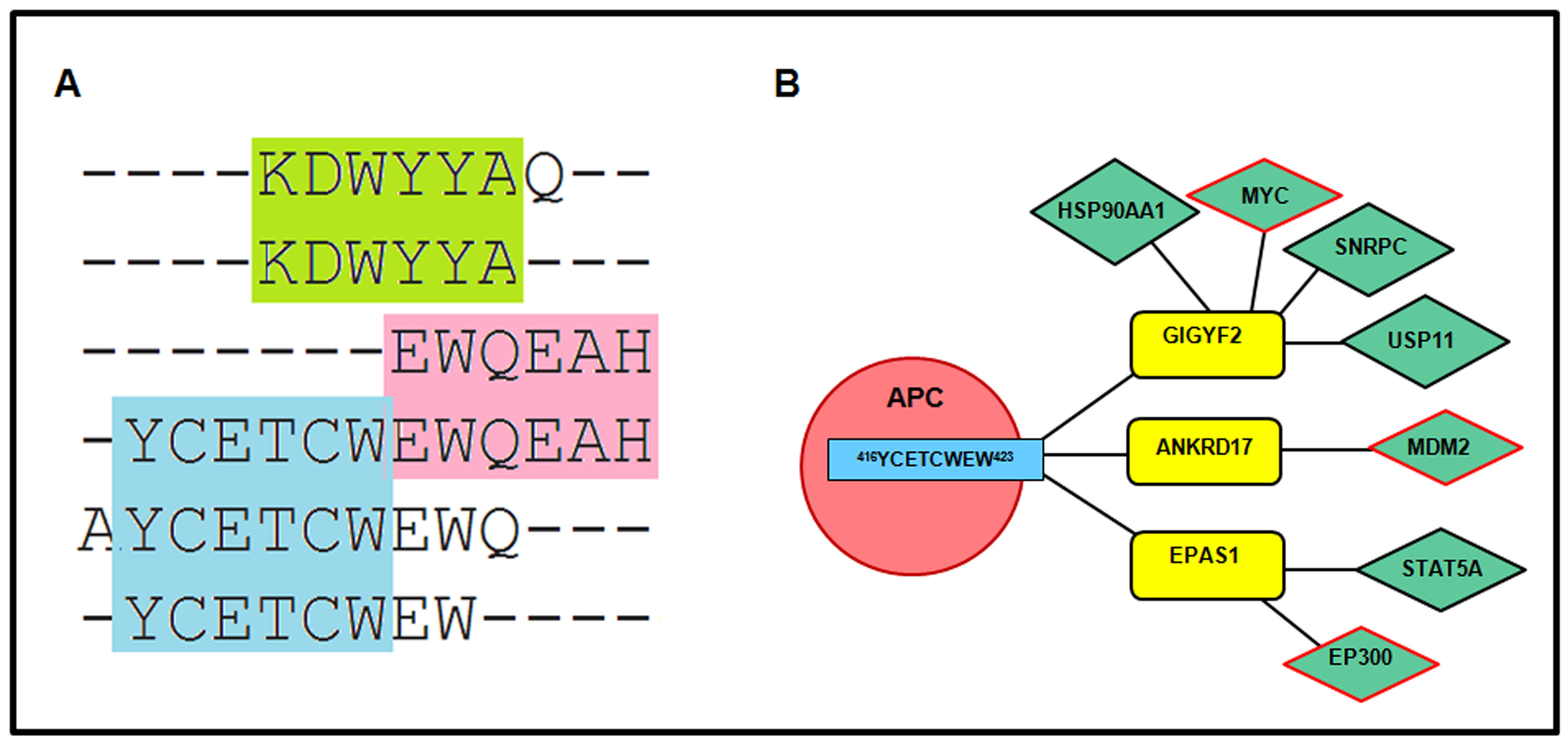
**

**Fig E**

**
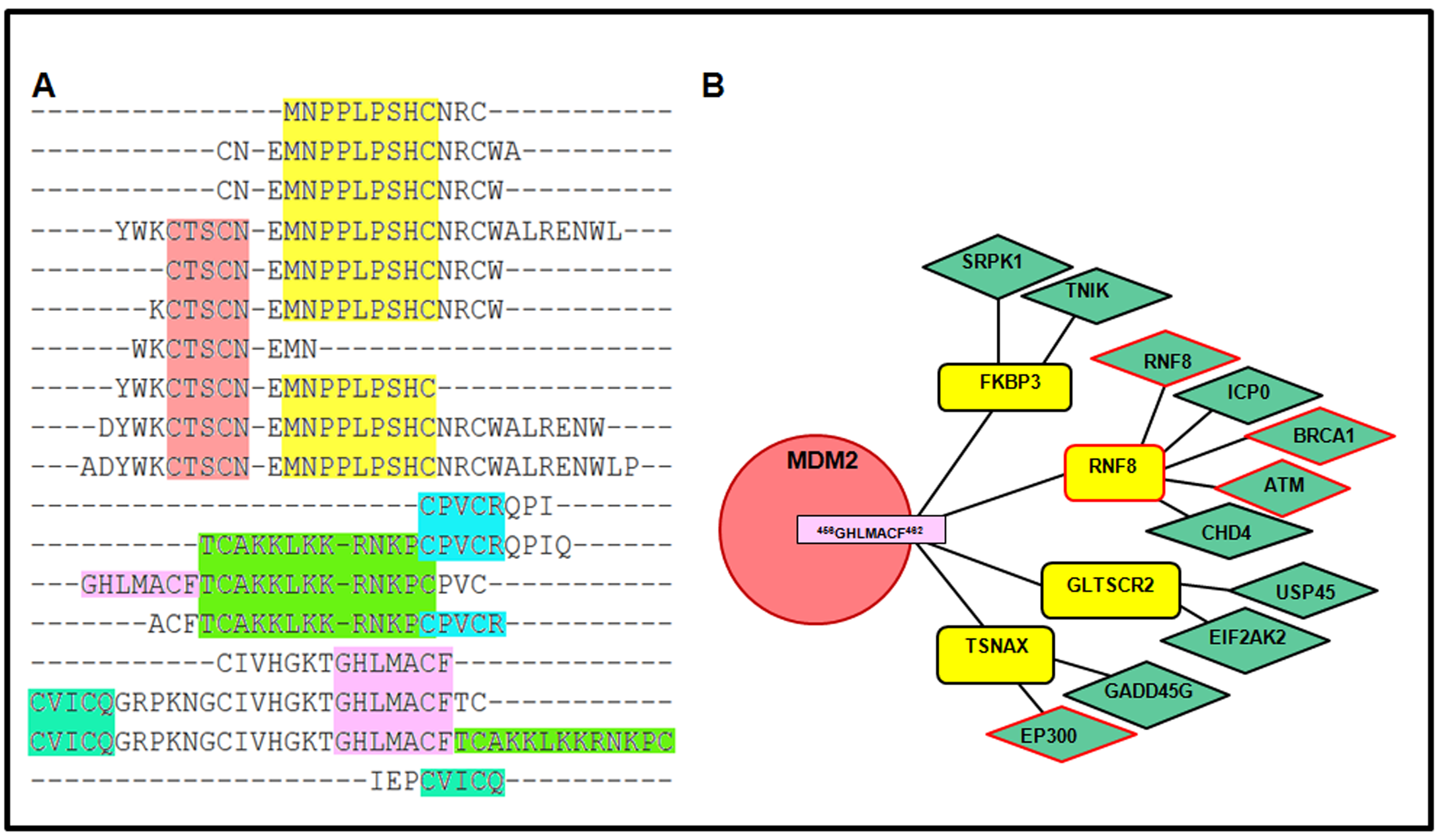
**

**Fig F**

**
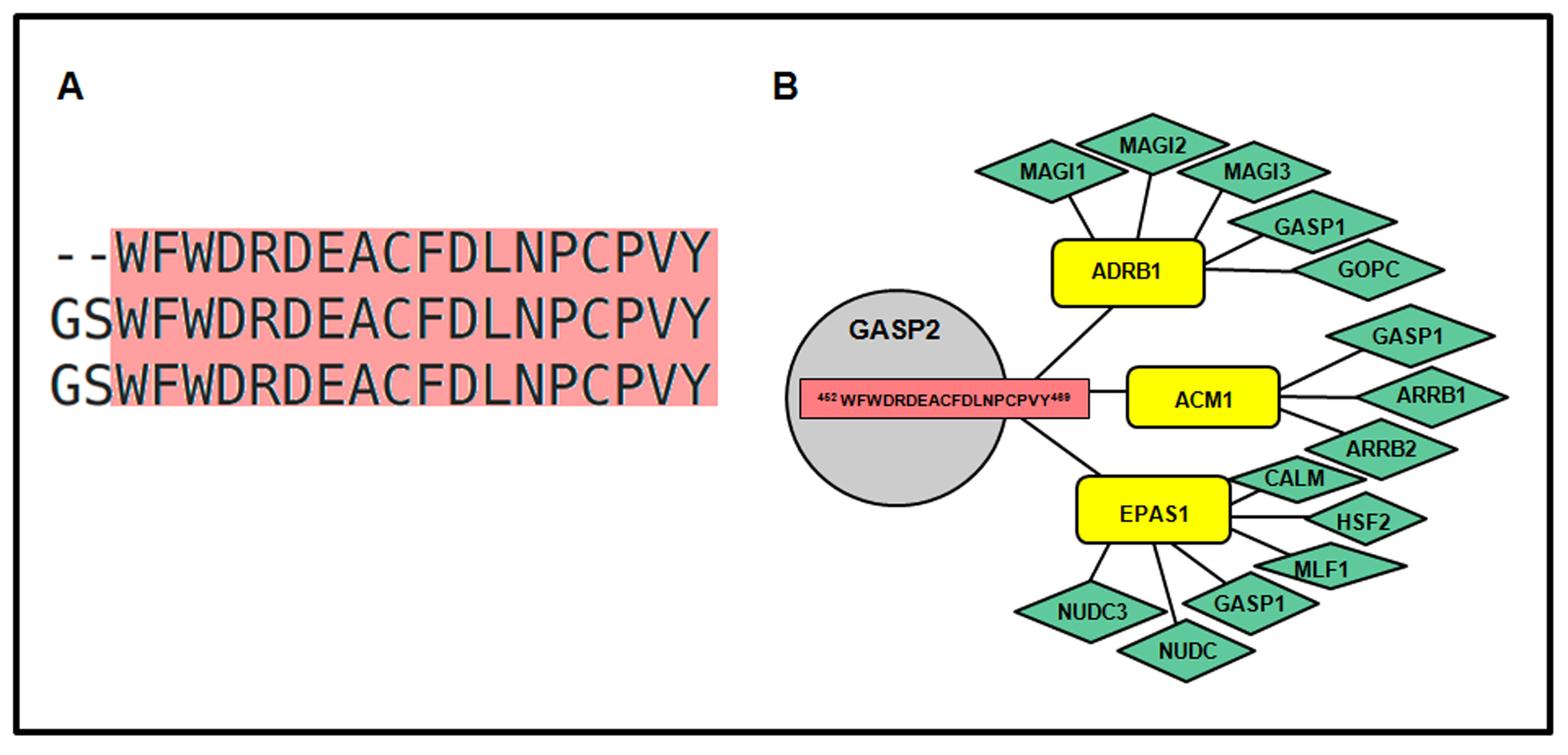
**

**Fig G**

**Screenshots of peptide-protein interactions predicted by PepSite2 server**

**(i) Images for peptides from MYC_HUMAN:**


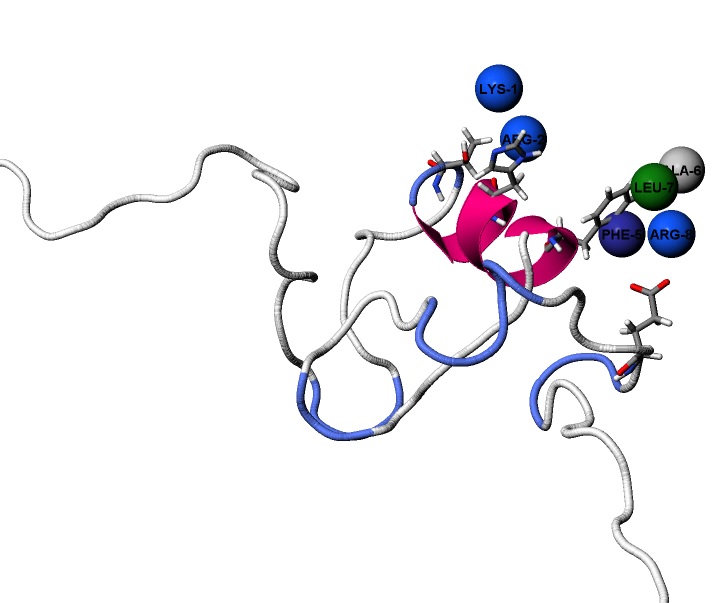


**Fig H1: Interaction of OLP - “371-KRSFFALRD-379” and FHPI - CNOT4 (p-value: 0.1576).**

**
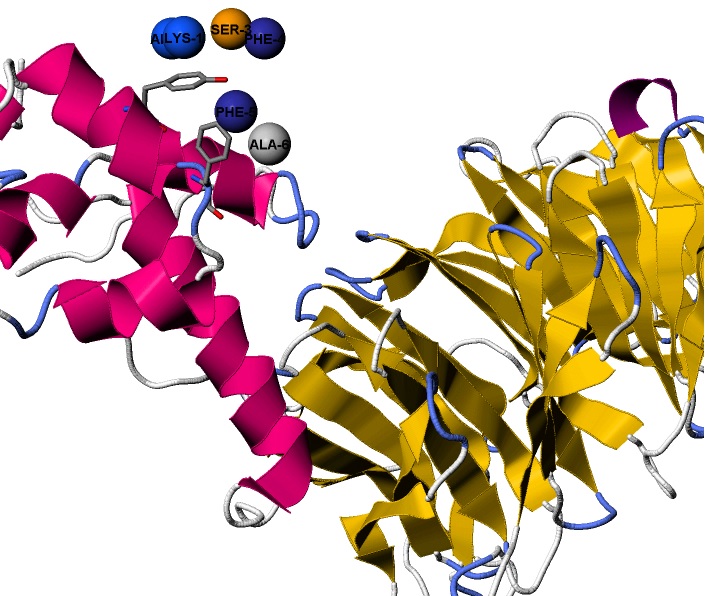
**

**Fig H2: Interaction of OLP - “371-KRSFFALRD-379” and FHPI - FBXW7 (p-value: 0.09191).**

**
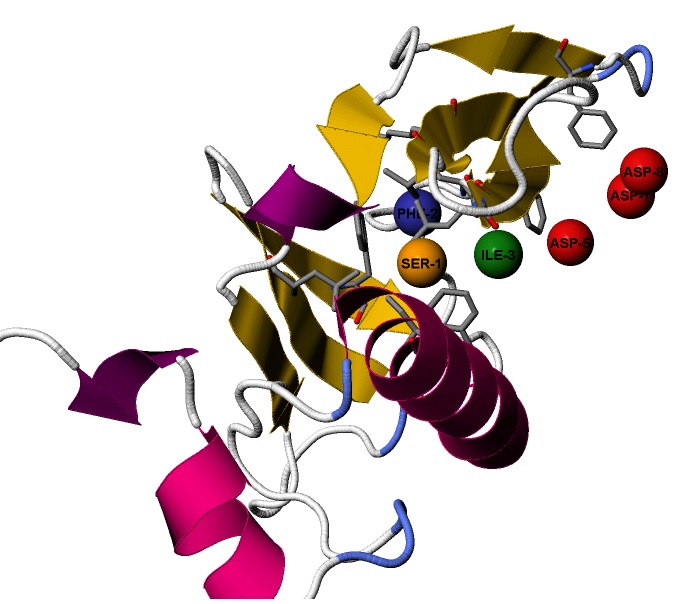
**

**Fig H3: Interaction of OLP - “114-SFICDPDD-121” and FHPI - EXOC1 (p-value: 0.2623).**

**
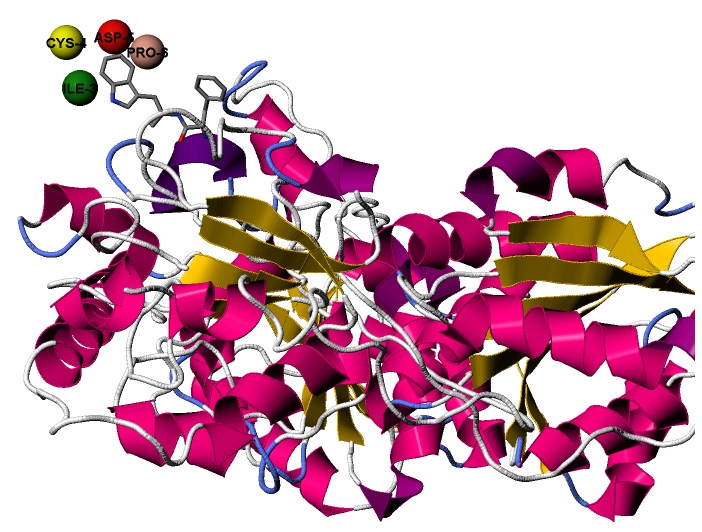
**

**Fig H4: Interaction of OLP - “114-SFICDPDD-121” and FHPI – ILVBL (p-value: 0.2637).**

**
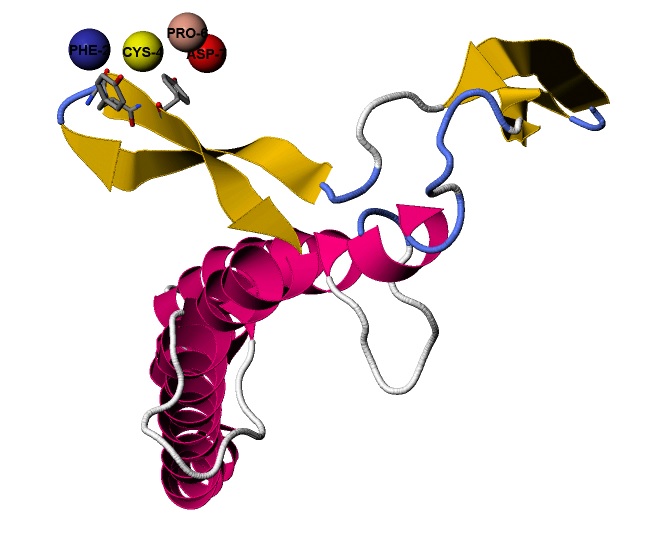
**

**Fig H5: Interaction of OLP - “114-SFICDPDD-121” and FHPI - PFDN5 (p-value: 0.4178).**

**
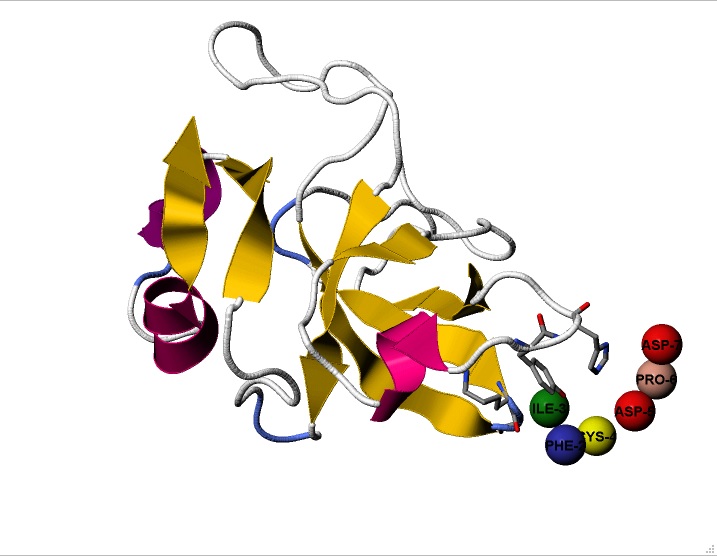
**

**Fig H6: Interaction of OLP- “114-SFICDPDD-121” and FHPI - MRPL14 (p-value: 0.3578).**

**
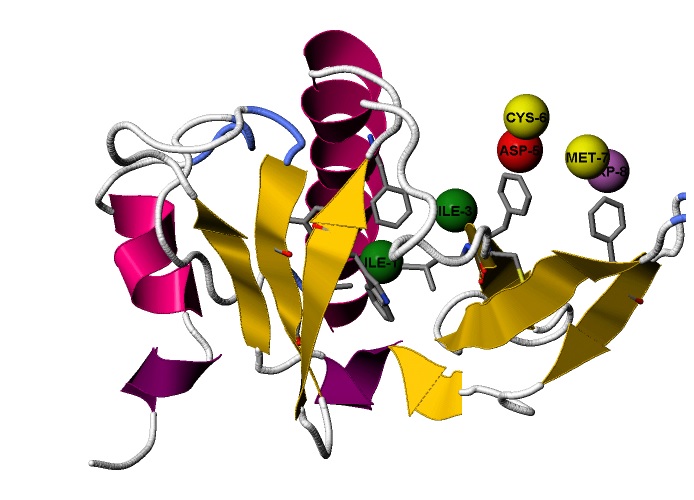
**

**Fig H7: Interaction of OLP - “128-IIIQDCMW-135” and FHPI - EXOC1 (p-value: 0.1183).**

**
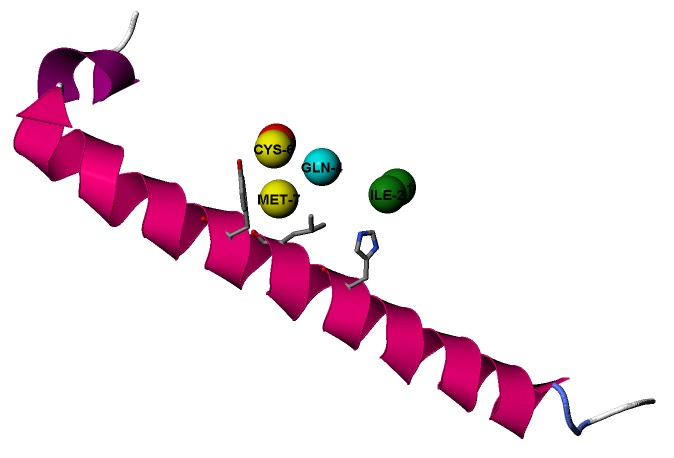
**

**Fig H8: Interaction of OLP - “128-IIIQDCMW-135” and FHPI - RAB11FIP5 (p-value: 0.2192).**

**
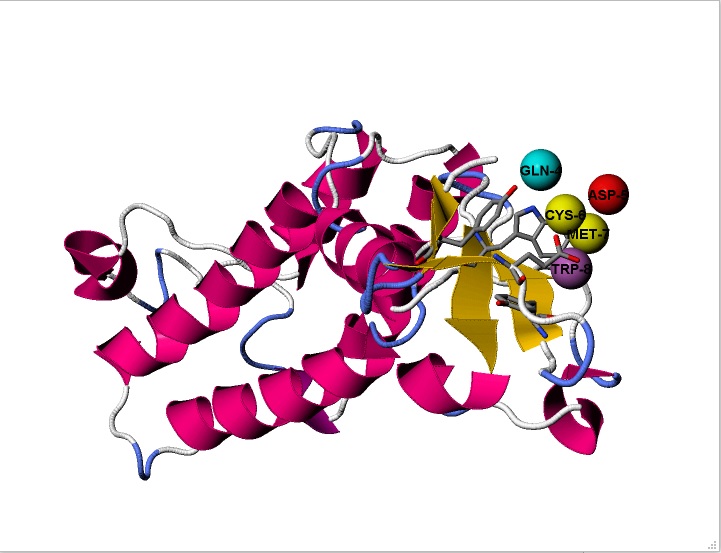
**

**Fig H9: Interaction of OLP - “128-IIIQDCMW-135” and FHPI - BPTF (p-value: 0.03816).**

**
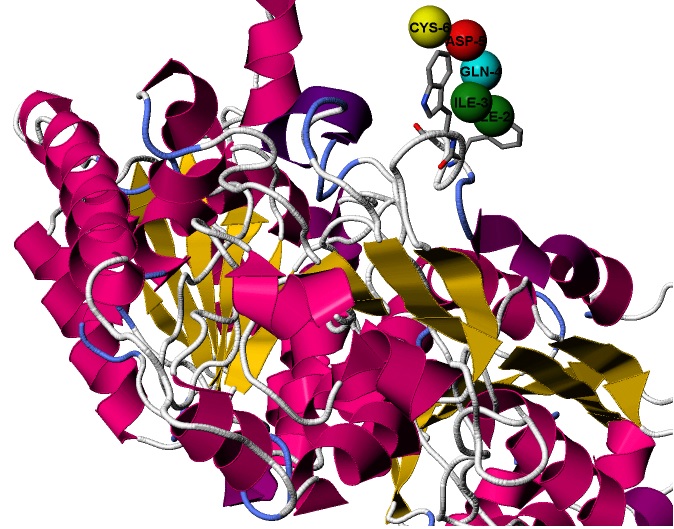
**

**Fig H10: Interaction of OLP - “128-IIIQDCMW-135” and FHPI – ILVBL (p-value: 0.1056).**

**
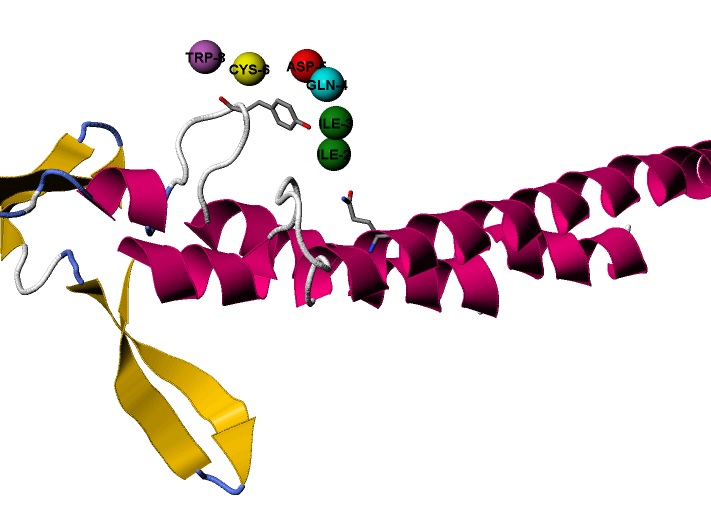
**

**Fig H11: Interaction of OLP - “128-IIIQDCMW-135” and FHPI - PFDN5 (p-value: 0.05528).**

**
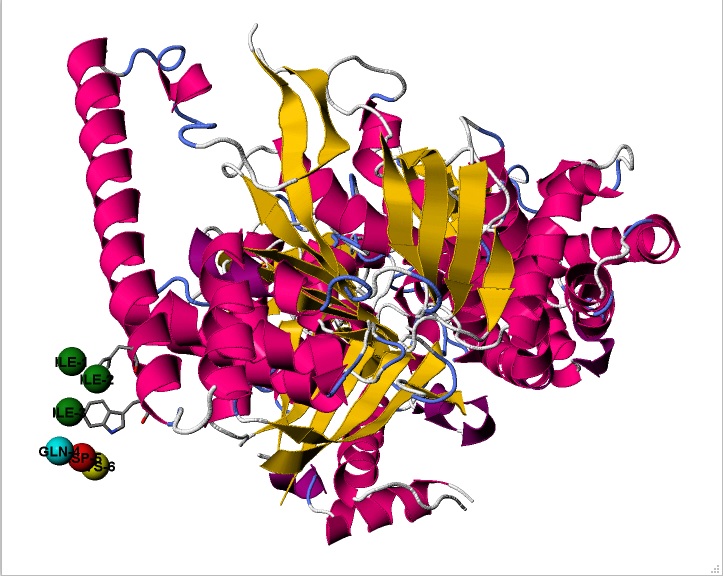
**

**Fig H12: Interaction of OLP - “128-IIIQDCMW-135” and FHPI - MSH3 (p-value: 0.02065).**

**
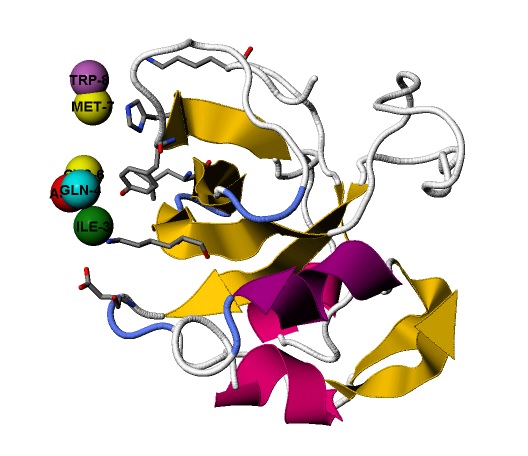
**

**Fig H13: Interaction of OLP - “128-IIIQDCMW-135” and FHPI - MRPL14 (p-value: 0.1401).**

**
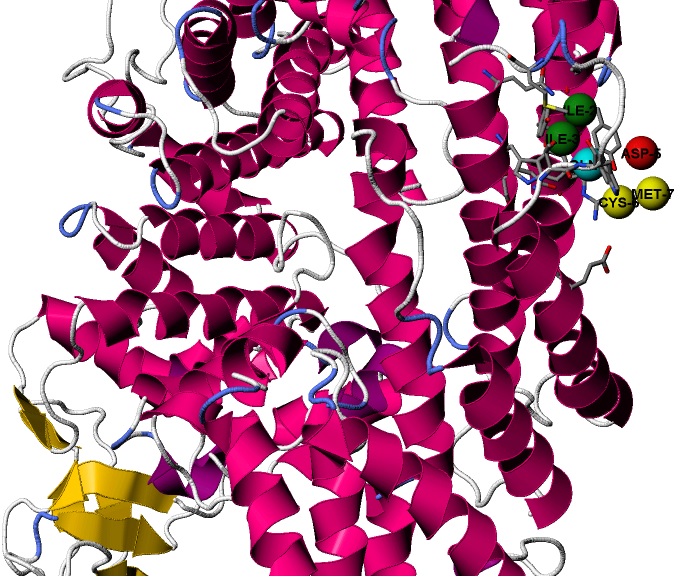
**

**Fig H14: Interaction of OLP - “128-IIIQDCMW-135” and FHPI - NUP188 (p-value: 0.001193).**

**
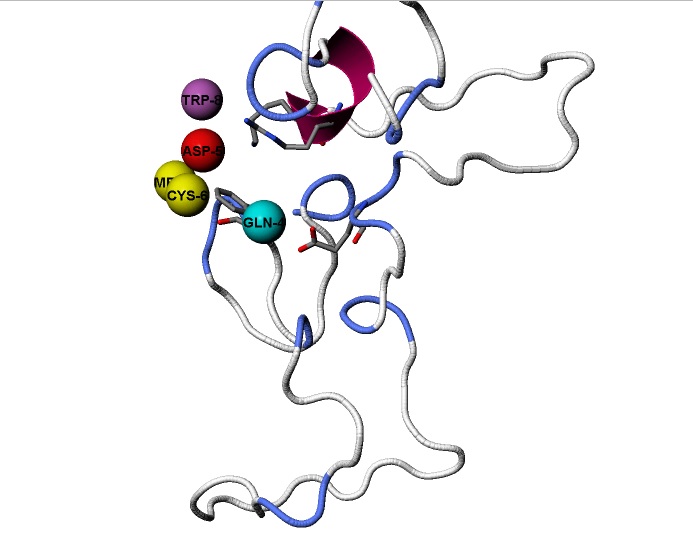
**

**Fig H15: Interaction of OLP - “128-IIIQDCMW-135” and FHPI - ZCCHC11 (p-value: 0.448).**

**
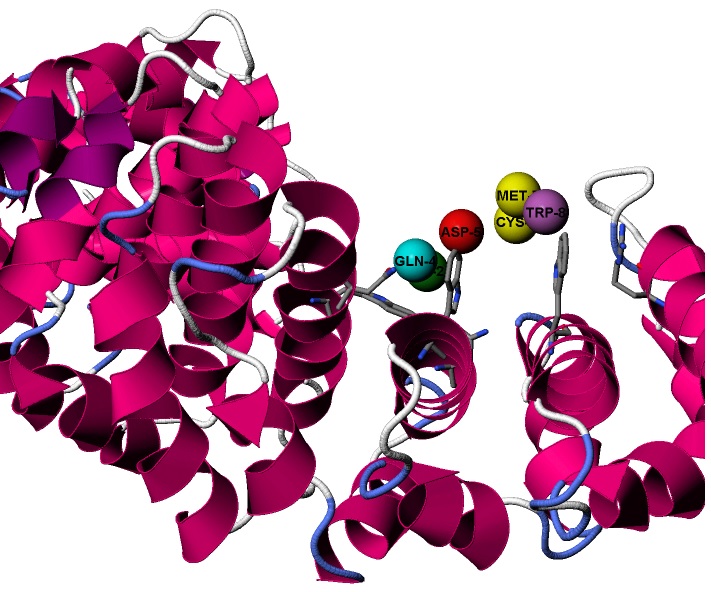
**

**Fig H16: Interaction of OLP - “128-IIIQDCMW-135” and FHPI- KPNA4 (p-value: 0.002434).**

**
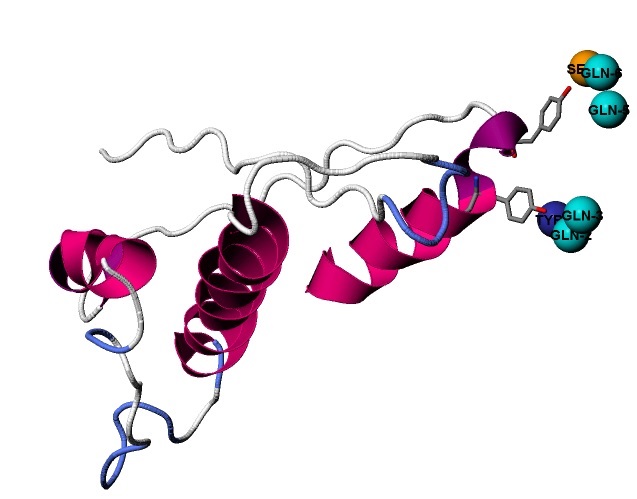
**

**Fig H17: Interaction of OLP - “32-YQQQQQSELQ-41” and FHPI - KIF20B (p-value: 0.05348).**

**
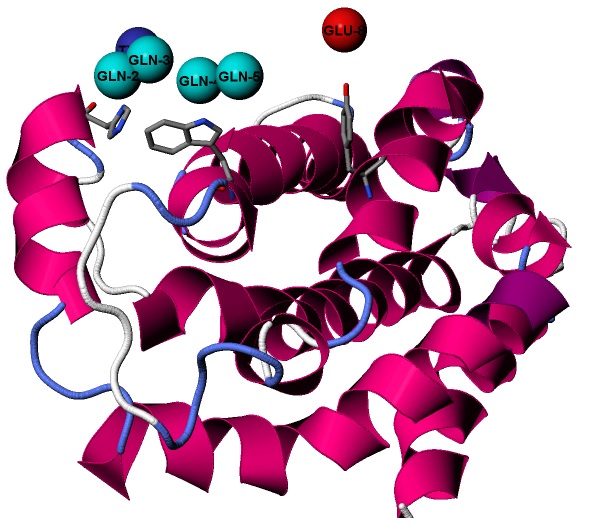
**

**Fig H18: Interaction of OLP - “32-YQQQQQSELQ-41” and FHPI – KALRN (p-value: 0.03485).**

**
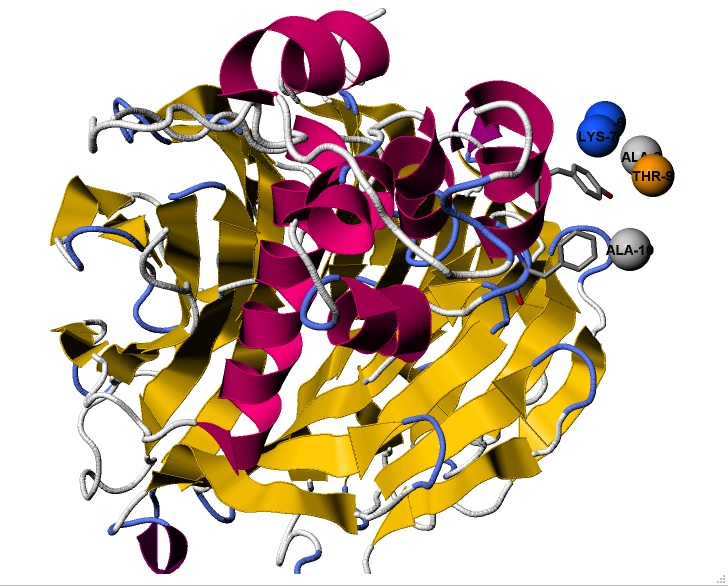
**

**Fig H19: Interaction of OLP-“392-KVVILKKATAY-402” and FHPI- FBXW7 (p-value: 0.3029).**

**
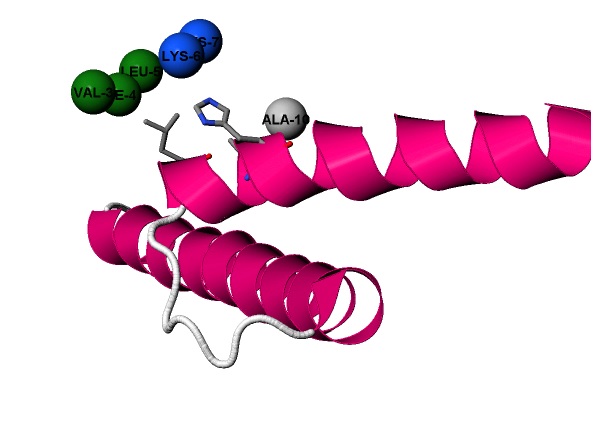
**

**Fig H20: Interaction of OLP-“392-KVVILKKATAY-402” and FHPI - TCF12 (p-value: 0.8503).**

**
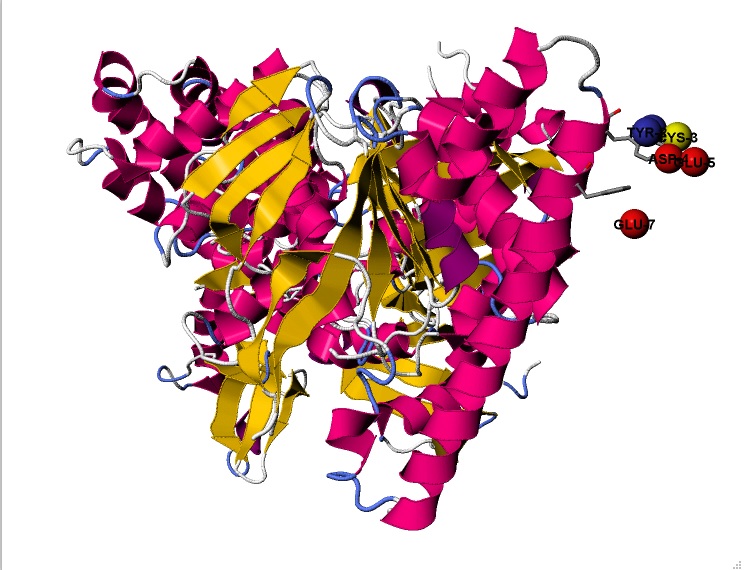
**

**Fig H21: Interaction of OLP - “23-FYCDEEEN-30” and FHPI - MSH3 (p-value: 0.06885).**

**
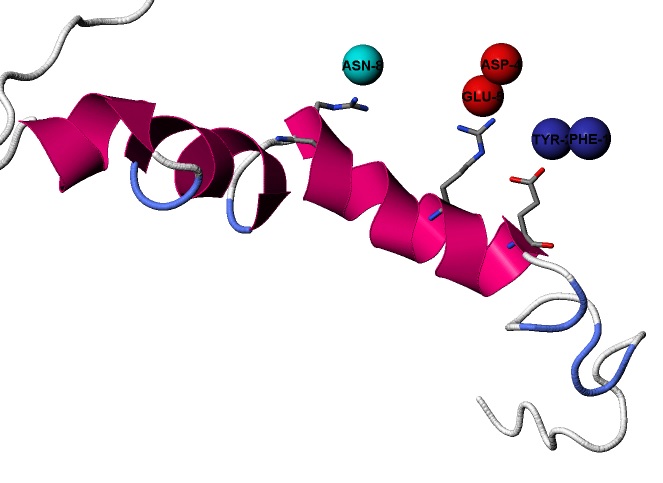
**

**Fig H22: Interaction of OLP - “23-FYCDEEEN-30” and FHPI - GIGYF2 (p-value: 0.9977).**

**
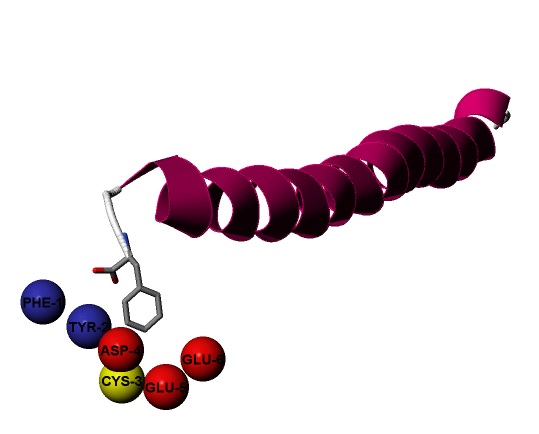
**

**Fig H23: Interaction of OLP - “23-FYCDEEEN-30” and FHPI - NFIL3 (p-value: 0.5086).**

**
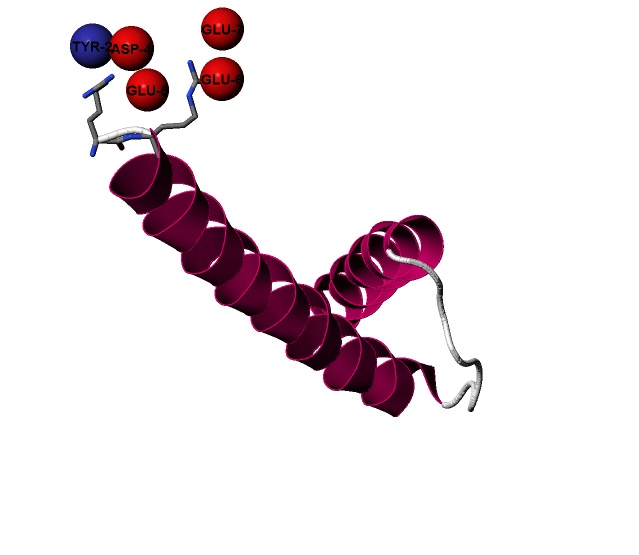
**

**Fig H24: Interaction of OLP - “23-FYCDEEEN-30” and FHPI - TCF12 (p-value: 0.9332).**

**
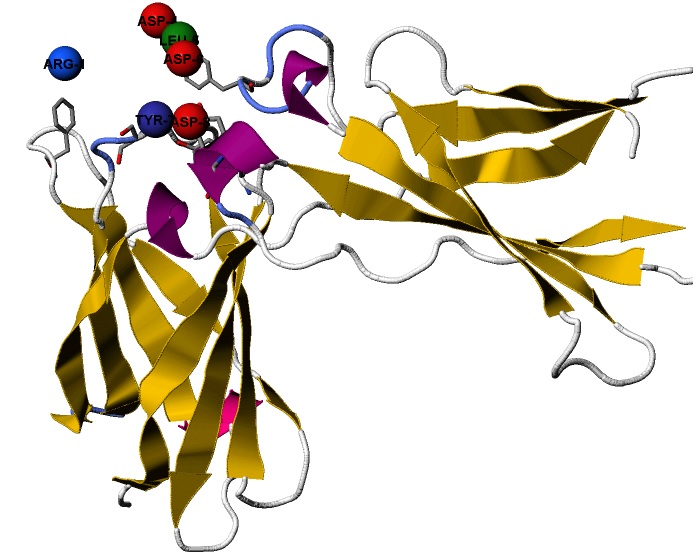
**

**Fig H25: Interaction of OLP - “10-RNYDLDYD-17” and FHPI - IL4R (p-value: 0.1391).**

**
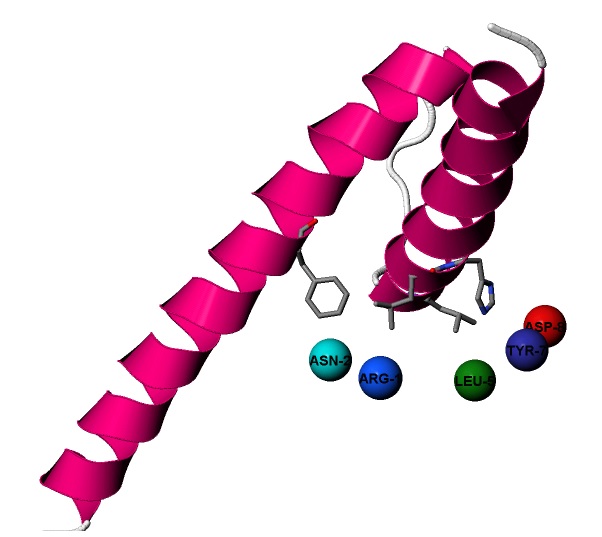
**

**Fig H26: Interaction of OLP - “10-RNYDLDYD-17” and FHPI - TCF12 (p-value: 0.9304).**

**
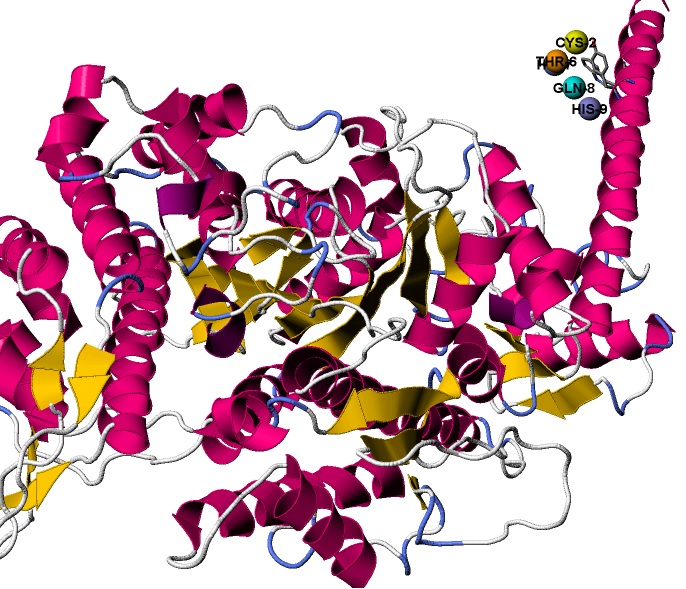
**

**Fig H27: Interaction of OLP - “10-RNYDLDYD-17” and FHPI - MYO1B (p-value: 0.1681).**

**(ii) Images for peptides from APC_HUMAN:**

**
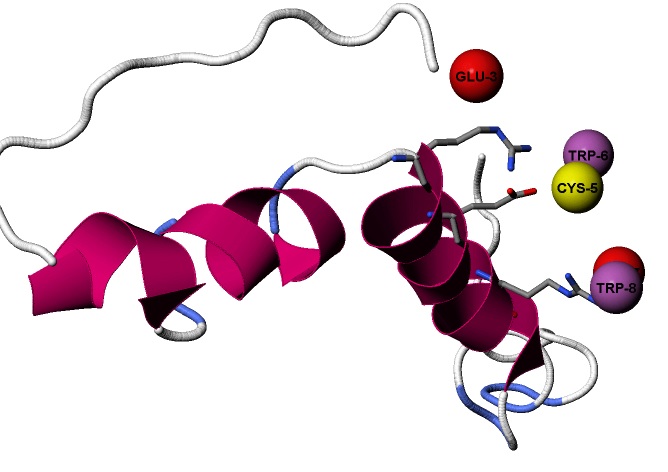
**

**Fig H28: Interaction of OLP - “416-YCETCWEW-423” and FHPI - GIGYF2 (p-value: 0.9374).**

**
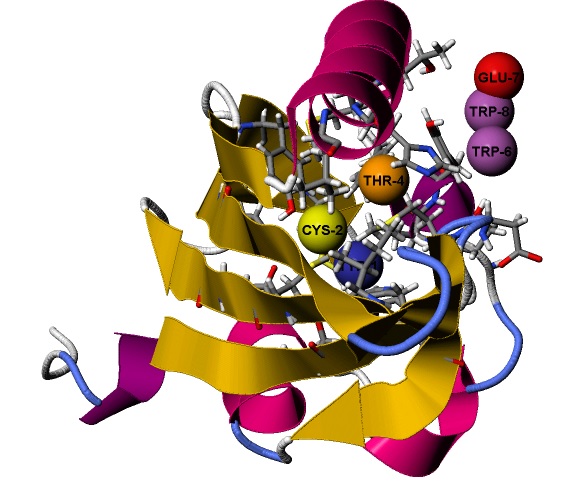
**

**Fig H29: Interaction of OLP - “416-YCETCWEW-423” and FHPI - EPAS1 (p-value: 0.1145).**

**
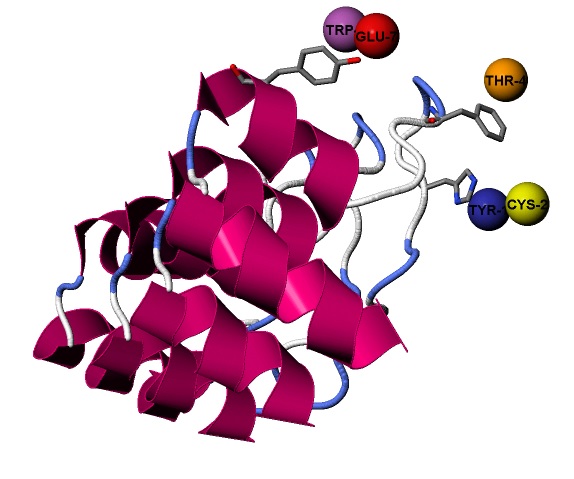
**

**Fig H30: Interaction of OLP - “416-YCETCWEW-423” and FHPI - ANKRD17 (p-value: 0.5264).**

**
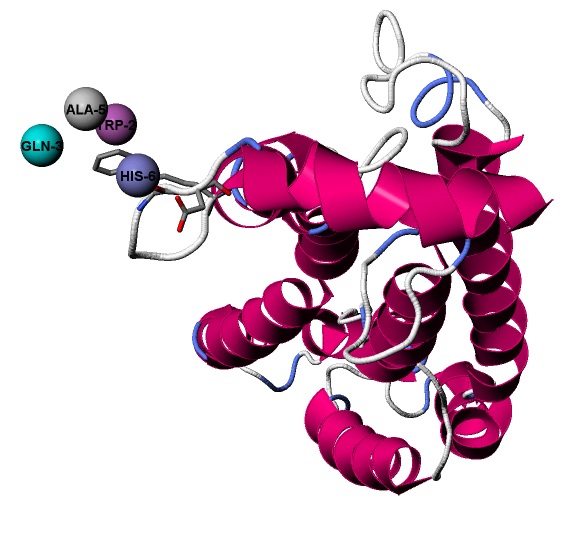
**

**Fig H31: Interaction of OLP - “422-EWQEAH-427” and FHPI - NCKAP5 (p-value: 0.4864).**

**
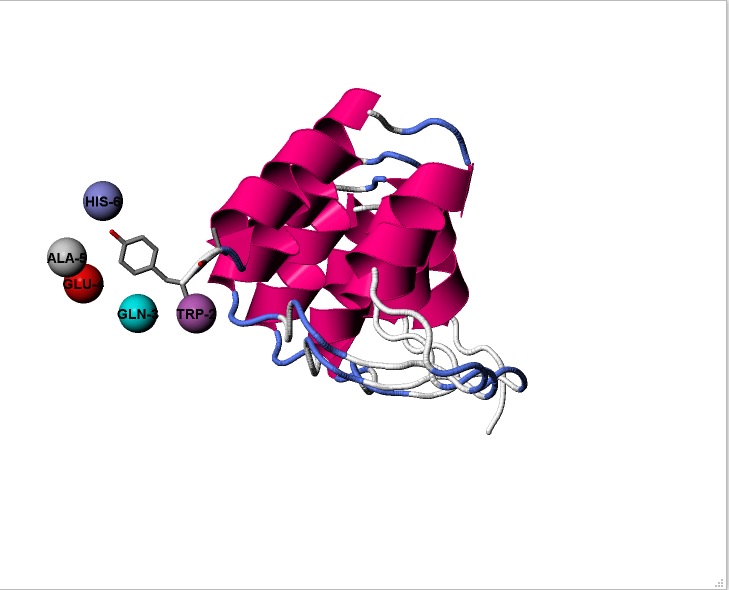
**

**Fig H32: Interaction of OLP - “422-EWQEAH-427” and FHPI - ANKRD17 (p-value: 0.2433).**

**
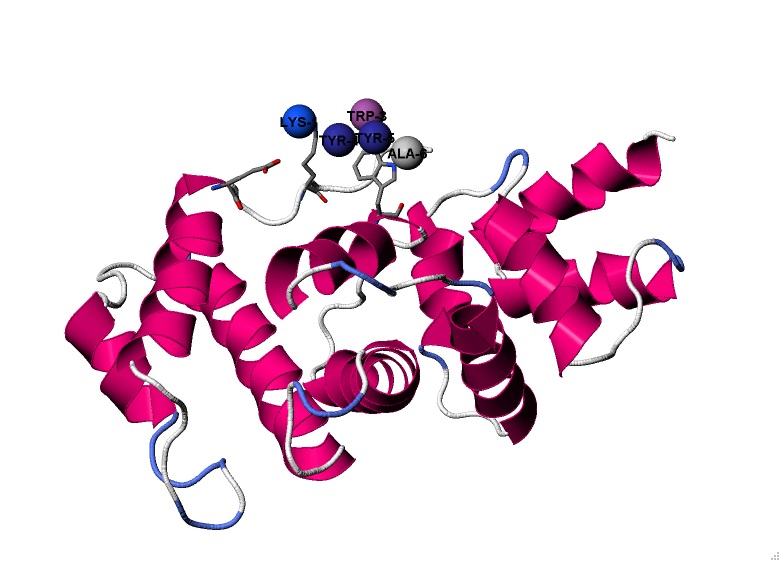
**

**Fig H33: Interaction of OLP - “155-KDWYYA-160” and FHPI - CYTH2 (p-value: 0.3792).**

**
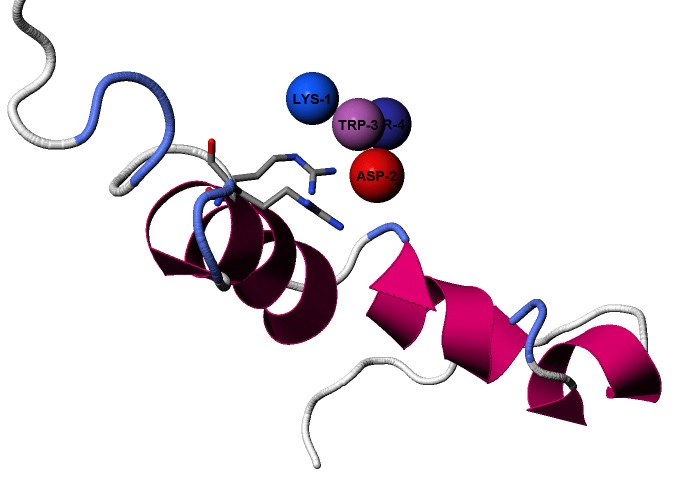
**

**Fig H34: Interaction of OLP - “155-KDWYYA-160” and FHPI - GIGYF2 (p-value: 0.993).**

**(iii) Images for peptides from MDM2_HUMAN:**

**
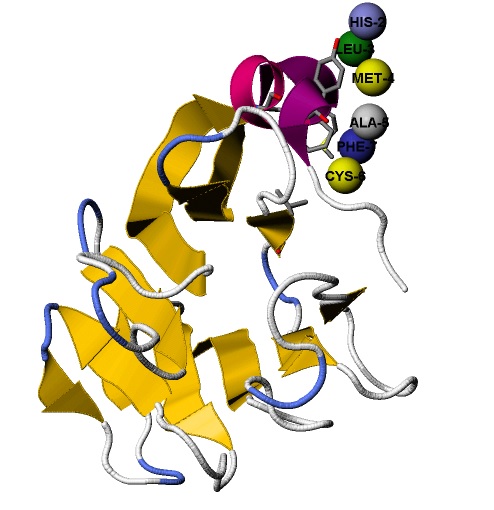
**

**Fig H35: Interaction of OLP - “456-GHLMACF-462” and FHPI - RNF8 (p-value: 0.01761).**

**
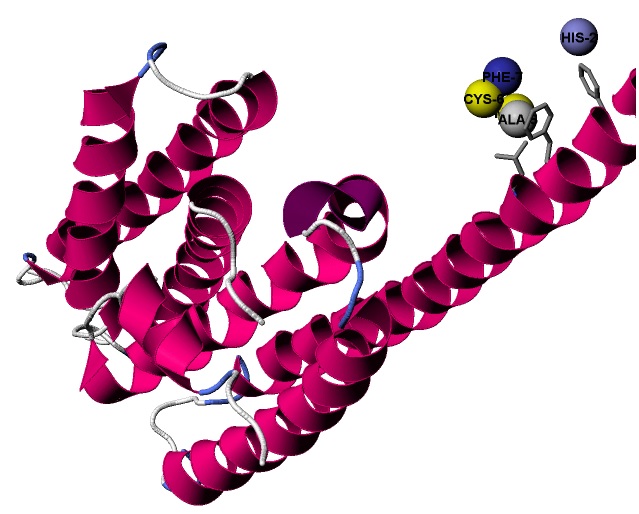
**

**Fig H36: Interaction of OLP - “456-GHLMACF-462” and FHPI – TSNAX (p-value: 0.1709).**

**
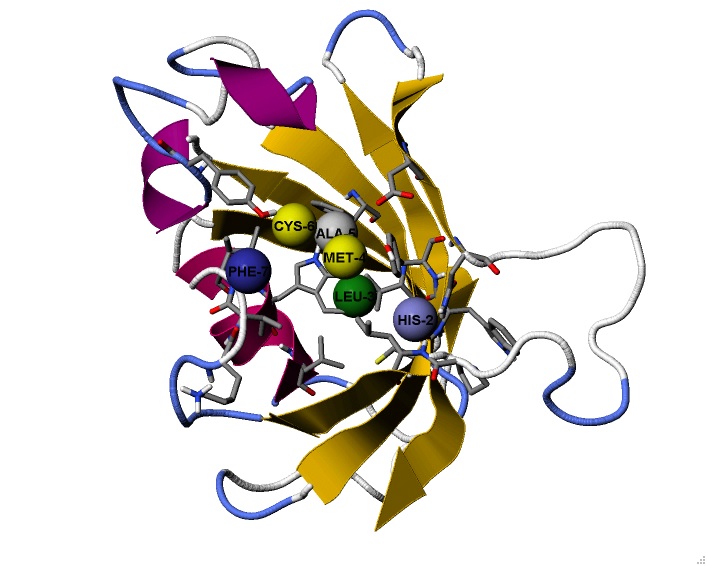
**

**Fig H37: Interaction of OLP - “456-GHLMACF-462” and FHPI - FKBP3 (p-value: 0.01931).**

**
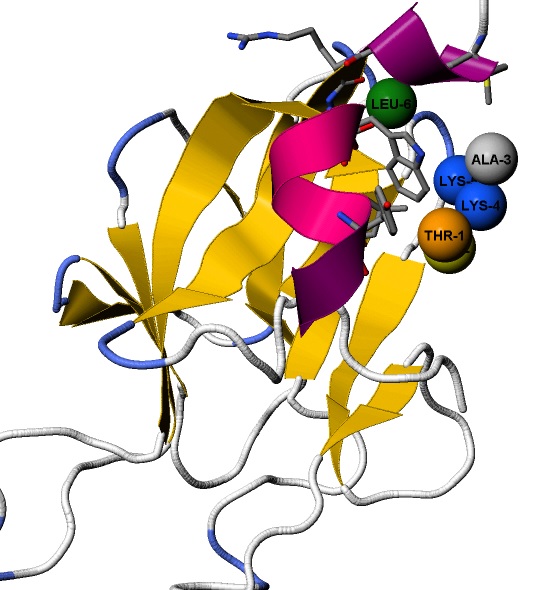
**

**Fig H38: Interaction of OLP-“463-TCAKKLKKRNKPC-475” and FHPI- RNF8 (p-value: 0.07304).**

**
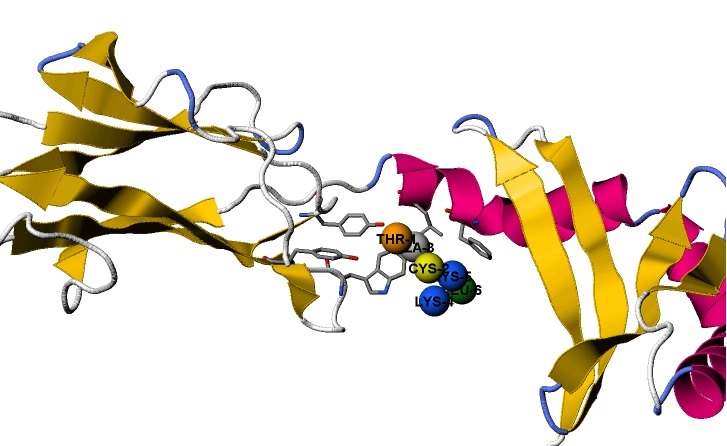
**

**Fig H39: Interaction of OLP - “463-TCAKKLKKRNKPC-475” and FHPI - HLA-DMB (p-value: 0.01368).**

**
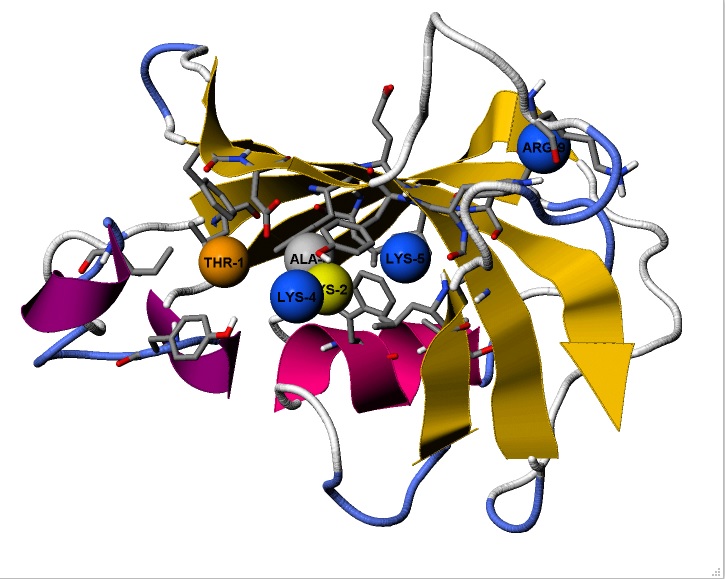
**

**Fig H40: Interaction of OLP - “463-TCAKKLKKRNKPC-475” and FHPI - FKBP3 (p-value: 0.1351).**

**
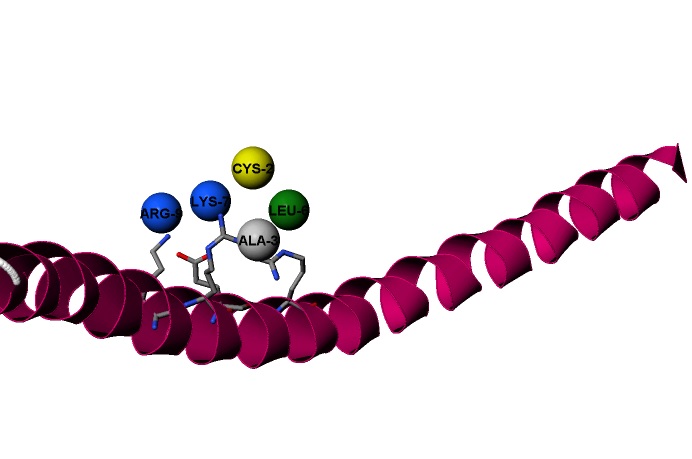
**

**Fig H41: Interaction of OLP - “463-TCAKKLKKRNKPC-475” and FHPI – JUND (p-value: 0.9997).**

**
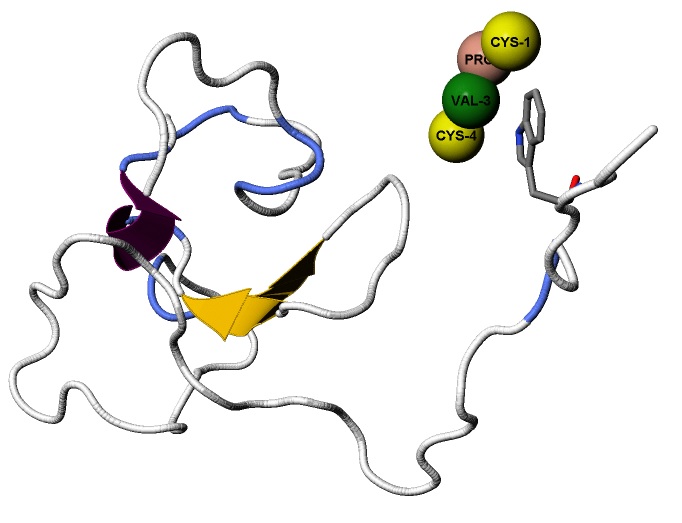
**

**Fig H42: Interaction of OLP - “475-CPVCR-478” and FHPI - PHF7 (p-value: 0.04128).**

**
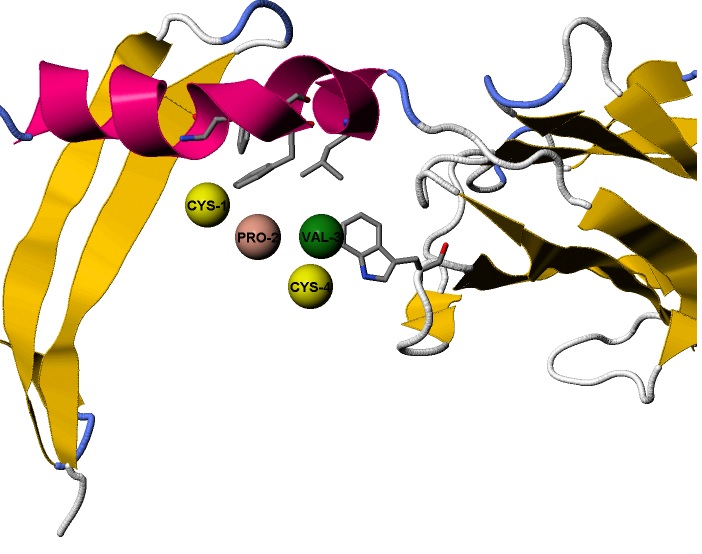
**

**Fig H43: Interaction of OLP - “475-CPVCR-478” and FHPI - HLA-DMB (p-value: 0.002625).**

**
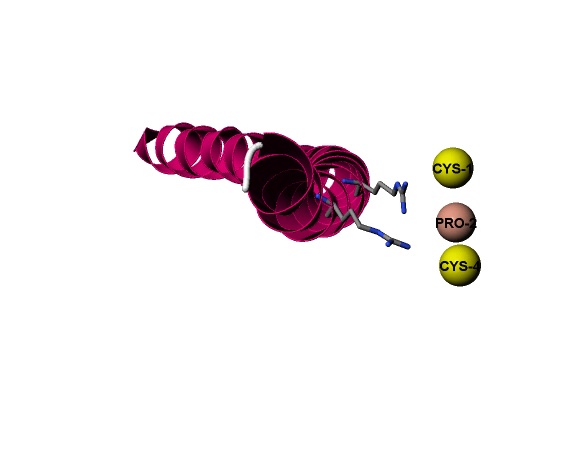
**

**Fig H44: Interaction of OLP - “475-CPVCR-478” and FHPI - JUND (p-value: 1.0).**

**
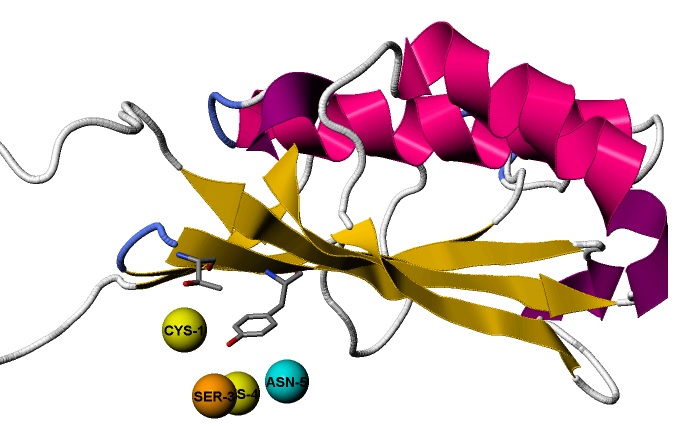
**

**Fig H45: Interaction of OLP - “305-CTSCN-309” and FHPI - HRSP12 (p-value: 0.3474).**

**
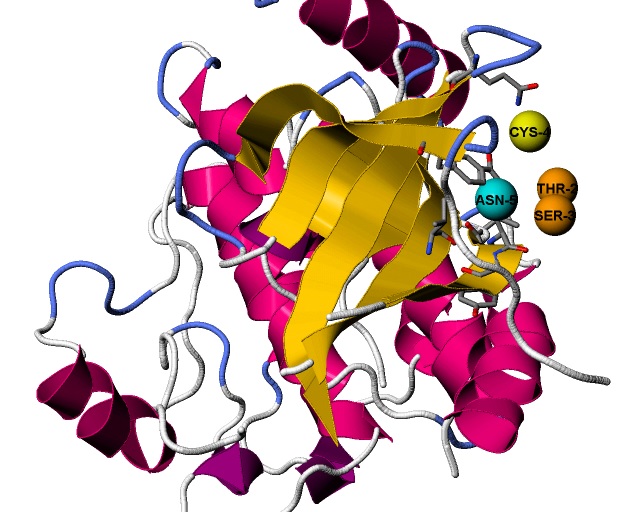
**

**Fig H46: Interaction of OLP - “305-CTSCN-309” and FHPI - MAP4K4 (p-value: 0.2628).**

**
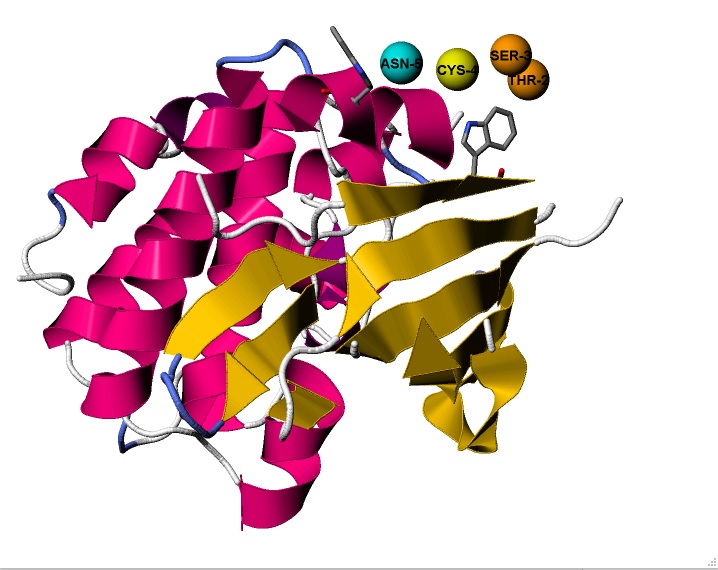
**

**Fig H47: Interaction of OLP - “305-CTSCN-309” and FHPI - PIM2 (p-value: 0.06501).**

**
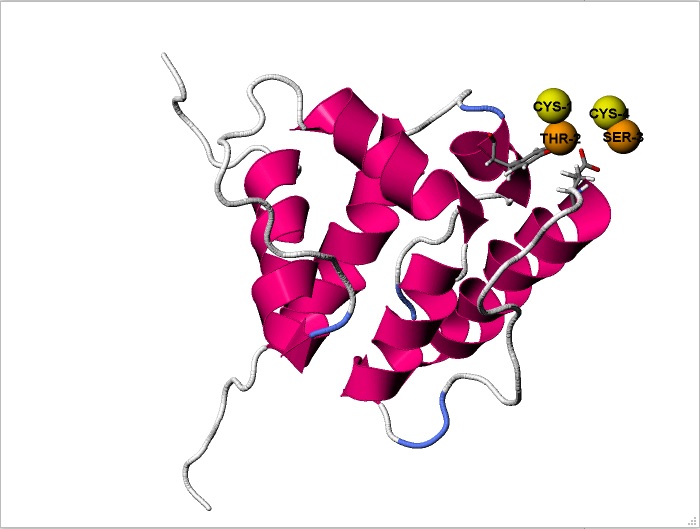
**

**Fig H48: Interaction of OLP-“305-CTSCN-309” and FHPI- ARHGEF6 (p-value: 0.005455).**

**
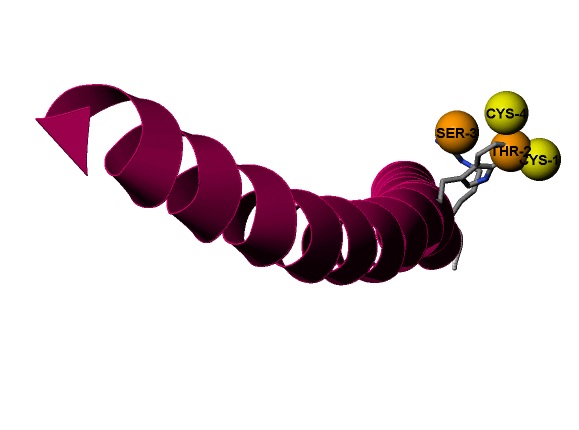
**

**Fig H49: Interaction of OLP - “305-CTSCN-309” and FHPI - NEFM (p-value: 0.2871).**

**
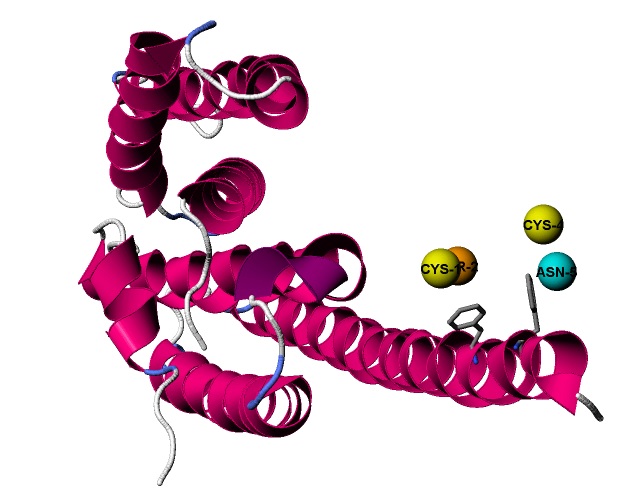
**

**Fig H50: Interaction of OLP - “305-CTSCN-309” and FHPI – TSNAX (p-value: 0.06092).**

**
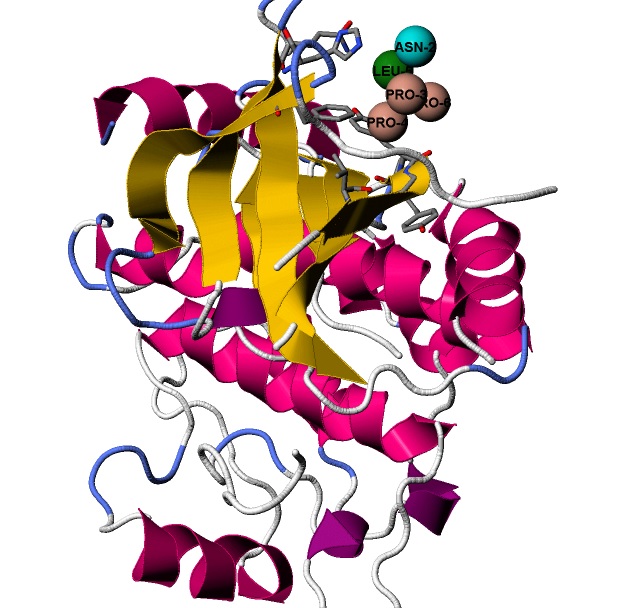
**

**Fig H51: Interaction of OLP - “311-MNPPLPSHC-319” and FHPI- MAP4K4 (p-value: 0.07288).**

**
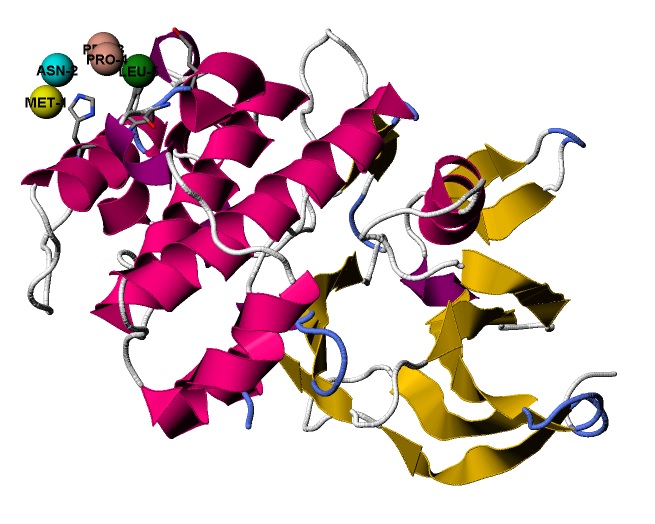
**

**Fig H52: Interaction of OLP - “311-MNPPLPSHC-319” and FHPI - PIM1 (p-value: 0.03425).**

**
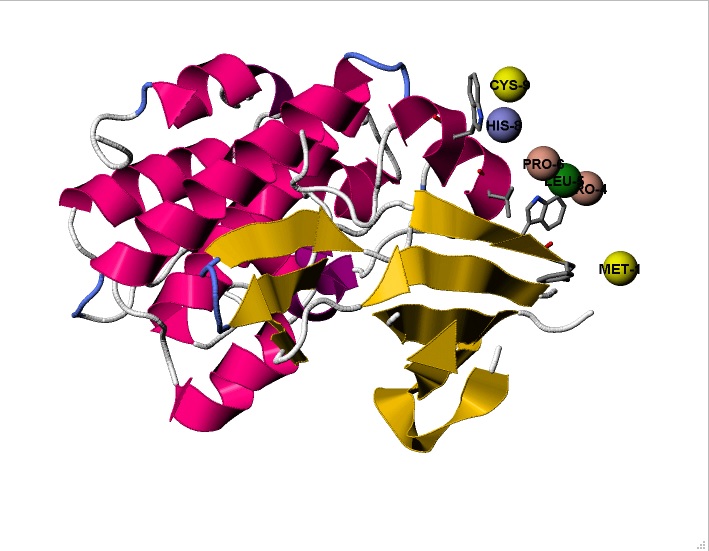
**

**Fig H53: Interaction of OLP - “311-MNPPLPSHC-319” and FHPI - PIM2 (p-value: 0.007897).**

**
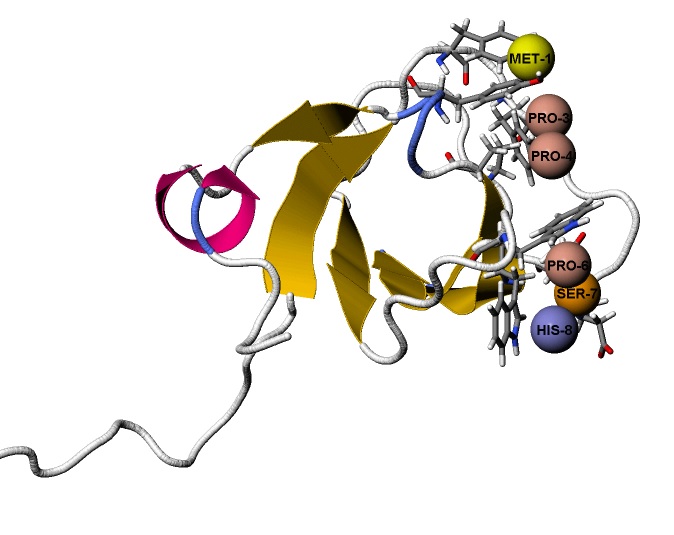
**

**Fig H54: Interaction of OLP-“311-MNPPLPSHC-319” and FHPI- ARHGEF6 (p-value: 9.897e-05).**

**
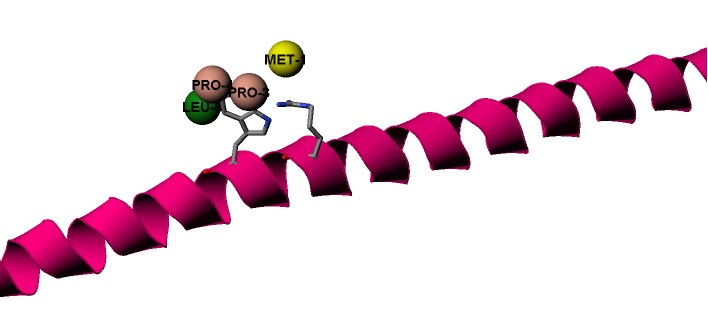
**

**Fig H55: Interaction of OLP - “311-MNPPLPSHC-319” and FHPI - NEFM (p-value: 0.4385).**

**
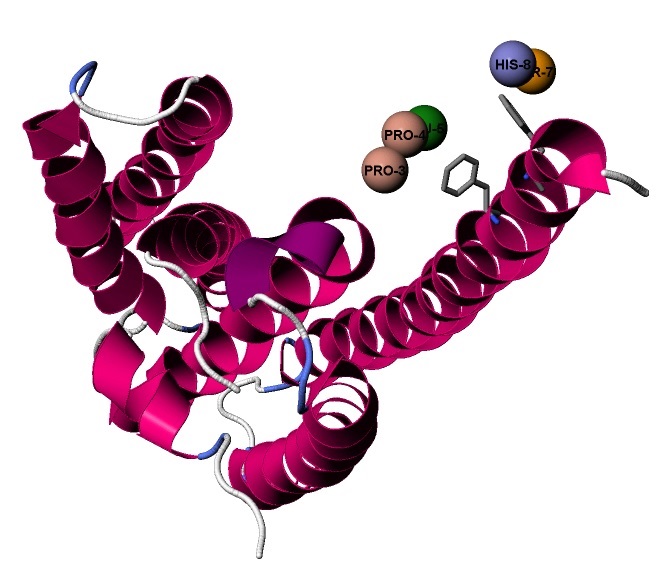
**

**Fig H56: Interaction of OLP - “311-MNPPLPSHC-319” and FHPI - TSNAX (p-value: 0.1849).**

**
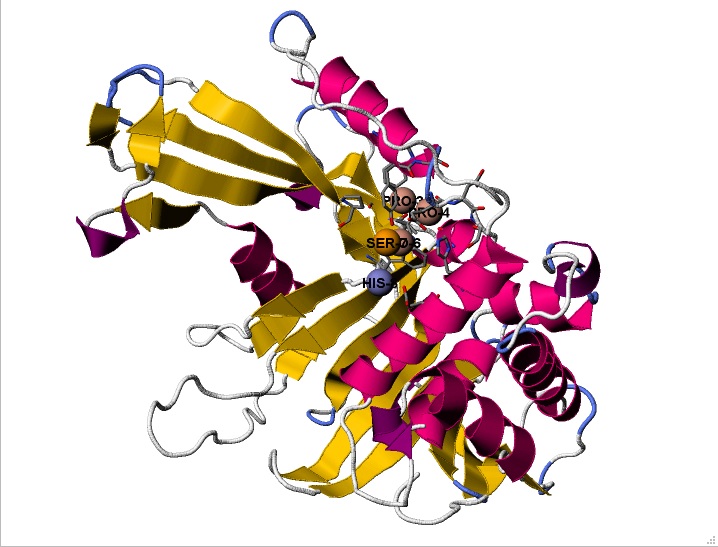
**

**Fig H57: Interaction of OLP-“311-MNPPLPSHC-319” and FHPI- USP2 (p-value: 0.1075).**

**
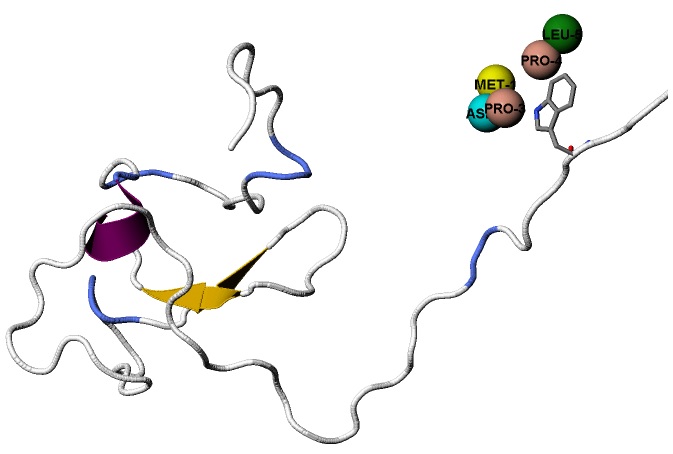
**

**Fig H58: Interaction of OLP - “438-CVICQ-442” and FHPI - PHF7 (p-value: 0.1627).**

**
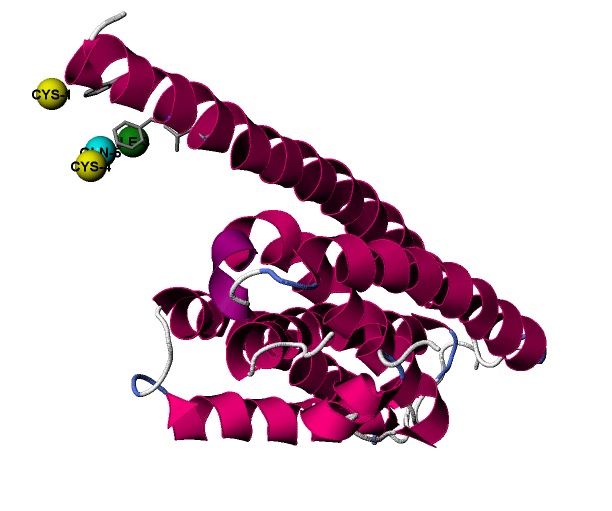
**

**Fig H59: Interaction of OLP - “438-CVICQ-442” and FHPI – TSNAX (p-value: 0.02254).**

**
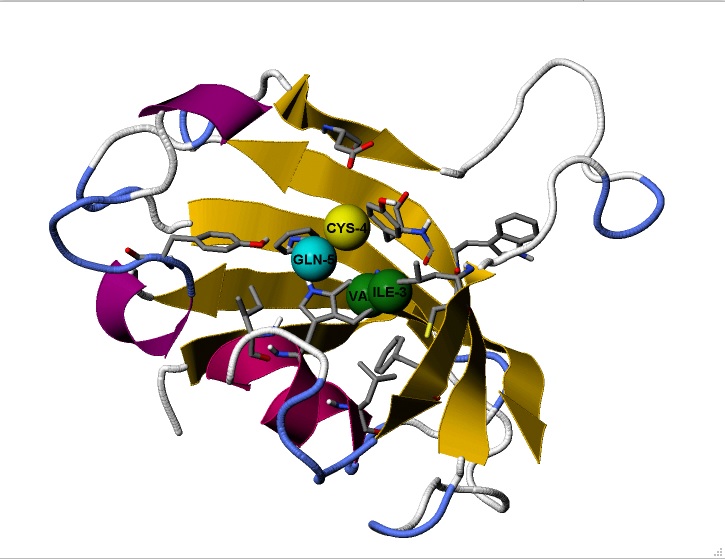
**

**Fig H60: Interaction of OLP - “438-CVICQ-442” and FHPI - FKBP3 (p-value: 0.01598).**

**Surface Accessibility predictions from SCRATCH prediction server:**

**(i) MYC_HUMAN:**

MPLNVSFTNRNYDLDYDSVQPYFYCDEEENFYQQQQQSELQPPAPSEDIWKKFELLPTPP

eee-e--e-ee-e-e-ee------eeeeee--e-eeeeeeeee---ee--ee-e-----e

LSPSRRSGLCSPSYVAVTPFSLRGDNDGGGGSFSTADQLEMVTELLGGDMVNQSFICDPD

---eeeeeee--ee-eee----eeeeeeeee--e---e-e---e--eeeeeeee-eeeee

DETFIKNIIIQDCMWSGFSAAAKLVSEKLASYQAARKDSGSPNPARGHSVCSTSSLYLQD

eeee-ee---e--------eeee-eeeeeeeeee-eeeeee-e-eeeeee-eeeee--e-

LSAAASECIDPSVVFPYPLNDSSSPKSCASQDSSAFSPSSDSLLSSTESSPQGSPEPLVL

-e-e--e--e-ee--e---eeeeeeee-e-eeeeeeeeeeeeeee-eeeeeeeeeeeeee

HEETPPTTSSDSEEEQEDEEEIDVVSVEKRQAPGKRSESGSPSAGGHSKPPHSPLVLKRC

eeeeeeeeeeeeeeeeeeeee------eeeeeeeeeeeeeeeeeeeeeeeeeeee--ee-

HVSTHQHNYAAPPSTRKDYPAAKRVKLDSVRVLRQISNNRKCTSPRSSDTEENVKRRTHN

e-eeeeee---eeeeeeeeeeeeeeeeeeeeeeeeeeeeeeeeeeeeeeeeeeeeeeeee

VLERQRRNELKRSFFALRDQIPELENNEKAPKVVILKKATAYILSVQAEEQKLISEEDLL

eeeeeeeee-ee--ee--e--ee-eeeee-ee-e--ee--e--ee-eee-ee-eee-ee-

RKRREQLKHKLEQLRNSCA

-ee-ee-eee—eeeeeee

**Fig I(i): The result of SCRATCH server of MYC protein where the yellow color highlighted region represent OLP, “e” represents surface accessible residue and “-“ represents buried residue.**

**(ii) APC_HUMAN:**

MAAASYDQLLKQVEALKMENSNLRQELEDNSNHLTKLETEASNMKEVLKQLQGSIEDEAM

eeeeeeee-eee-ee-eee-ee-eee-eeeeee-ee-eeeeeeeeeeeeeeeeeeeeeee

ASSGQIDLLERLKELNLDSSNFPGVKLRSKMSLRSYGSREGSVSSRSGECSPVPMGSFPR

e-ee--e--e--ee-e-e-ee-ee-e-e-e-e-eeeeeeeeee-ee--eeee-e----ee

RGFVNGSRESTGYLEELEKERSLLLADLDKEEKEKDWYYAQLQNLTKRIDSLPLTENFSL

ee--ee-ee--e--ee-eee-----ee-eeeeeeee--ee--ee--ee-ee-ee-ee---

QTDMTRRQLEYEARQIRVAMEEQLGTCQDMEKRAQRRIARIQQIEKDILRIRQLLQSQAT

eee--eee-e-e-ee-ee--eeeee-eeee-eeeeee-eeeee-eee--e-eee-eeeee

EAERSSQNKHETGSHDAERQNEGQGVGEINMATSGNGQGSTTRMDHETASVLSSSSTHSA

eeeeeeeeeeeeeeeeeeeeeeeee-ee-e-----e-eeeeeeeeee-e-----eeeee-

PRRLTSHLGTKVEMVYSLLSMLGTHDKDDMSRTLLAMSSSQDSCISMRQSGCLPLLIQLL

eeeeeeeee---------------eeeee--e---e--eeee------e-----------

HGNDKDSVLLGNSRGSKEARARASAALHNIIHSQPDDKRGRREIRVLHLLEQIRAYCETC
-eeeee-eeeeeeeeeee--ee--e---e--e-eeeeeeee---e--e--e---e--e--

WEWQEAHEPGMDQDKNPMPAPVEHQICPAVCVLMKLSFDEEHRHAMNELGGLQAIAELLQ

ee--eeeeeeeeeeeee-eee-ee----------e---eee--e------------e--e

VDCEMYGLTNDHYSITLRRYAGMALTNLTFGDVANKATLCSMKGCMRALVAQLKSESEDL

-e-e--e-eeee------------------e-ee------e-ee--e---e--eee-ee-

QQVIASVLRNLSWRADVNSKKTLREVGSVKALMECALEVKKESTLKSVLSALWNLSAHCT

-ee----------e-eee-ee--ee-e--e---e---e-eeee--e-----------e-e

ENKADICAVDGALAFLVGTLTYRSQTNTLAIIESGGGILRNVSSLIATNEDHRQILRENN

eee-e--e-ee-----------e-eeee-e-----------------eeee-ee--eeee

CLQTLLQHLKSHSLTIVSNACGTLWNLSARNPKDQEALWDMGAVSMLKNLIHSKHKMIAM

--e---e--eeeeee--------------e-ee--e---e-e--e--ee--e-eee---e

GSAAALRNLMANRPAKYKDANIMSPGSSLPSLHVRKQKALEAELDAQHLSETFDNIDNLS

------e---eeeeee-eeeee-eeee--------e-e--eee-e-e---e--ee-eee-

PKASHRSKQRHKQSLYGDYVFDTNRHDDNRSDNFNTGNMTVLSPYLNTTVLPSSSSSRG
-eeeeeeeeee-ee--ee---e----eeee-ee-eee-----------e--eee-eee—

LDSSRSEKDRSLERERGIGLGNYHPATENPGTSSKRGLQISTTAAQIAKVMEEVSAIHTS

-eee-eeeeeeeeeeeeeeeeeeeeeee-ee-eee--ee--e---e-ee--ee--e---e

QEDRSSGSTTELHCVTDERNALRRSSAAHTHSNTYNFTKSENSNRTCSMPYAKLEYKRSS

eeeeeeee-ee-e--eee-ee-eeee--ee-eee-e-eeeee-e---e-----e--eeee

NDSLNSVSSSDGYGKRGQMKPSIESYSEDDESKFCSYGQYPADLAHKIHSANHMDDNDGE

eee-e-----ee-eeeeeeeee-ee-eeeeeeee-eeee-eee--ee-ee-ee-eeee-e

LDTPINYSLKYSDEQLNSGRQSPSQNERWARPKHIIEDEIKQSEQRQSRNQSTTYPVYTE

eeeeee---e--eeeeeeeeeeeeeeee-eeeee--eee-eeeeeeeeeeeeee----ee

STDDKHLKFQPHFGQQECVSPYRSRGANGSETNRVGSNHGINQNVSQSLCQEDDYEDDKP

eeeeee-eeeee-eeeeeeeeeeeeeeeeeeeee-eeeee-eeeeee---eeeeeeeeee

TNYSERYSEEEQHEEEERPTNYSIKYNEEKRHVDQPIDYSLKYATDIPSSQKQSFSFSKS

eee-ee--eeeeeeeeeeeeee--eeeeeeeeeeee-e--ee--eeeeeeeeee-e-ee-

SSGQSSKTEHMSSSSENTSTPSSNAKRQNQLHPSSAQSRSGQPQKAATCKVSSINQETIQ

ee-ee-eeee-eeeeeeeeeeeeeeeeeeeee-ee-eeeeeeeeeeeeeeeee-eeee-e

TYCVEDTPICFSRCSSLSSLSSAEDEIGCNQTTQEADSANTLQIAEIKEKIGTRSAEDPV

e--eee------------e-eeeeeeeeeeeeeeeeeeeee-eeeeeeeeeeeeeeeeee

SEVPAVSQHPRTKSSRLQGSSLSSESARHKAVEFSSGAKSPSKSGAQTPKSPPEHYVQET

eeeeeeeeeeeeeeeeeeeee-eeeeeeeeeeeeeeeeeeeeeeeee-eeeeeee--ee-

PLMFSRCTSVSSLDSFESRSIASSVQSEPCSGMVSGIISPSDLPDSPGQTMPPSRSKTPP

-------ee-ee-ee-eeeeeeee-e-ee--ee------e-e-eeeeeee-ee-eeeeee

PPPQTAQTKREVPKNKAPTAEKRESGPKQAAVNAAVQRVQVLPDADTLLHFATESTPDGF

eeeeeeeeeeeeeeeeeeeeeeeeeeeeee--ee-eeeeee-eeee-----eeee-eee-

SCSSSLSALSLDEPFIQKDVELRIMPPVQENDNGNETESEQPKESNENQEKEAEKTIDSE

----e----e-eeeeeeee-eeeeeeeeeeeeeeeeeeeeeeeeeeeeeeeeeeee-eee

KDLLDDSDDDDIEILEECIISAMPTKSSRKAKKPAQTASKLPPPVARKPSQLPVYKLLPS

eeeeeeeeeee-e--ee---ee-eeeeeeeeeeeeeeeeeeeeeeeeeeee-e--e--ee

QNRLQPQKHVSFTPGDDMPRVYCVEGTPINFSTATSLSDLTIESPPNELAAGEGVRGGAQ

eeeeeeeee-eeeeeeee------ee------e-----e---e-eeeeeeeeee-eeeee

SGEFEKRDTIPTEGRSTDEAQGGKTSSVTIPELDDNKAEEGDILAECINSAMPKGKSHKP

eeeeeeeee-eeeeeeeeeeeeeeeeeee-eeeeeeeeeeee--ee--eee-eeeeeeee

FRVKKIMDQVQQASASSSAPNKNQLDGKKKKPTSPVKPIPQNTEYRTRVRKNADSKNNLN

eeeeee-ee-eeeeeeeee-eeeeee-eeeeee-eeee-eeeee-ee--eeeeeeeee--

AERVFSDNKDSKKQNLKNNSKVFNDKLPNNEDRVRGSFAFDSPHHYTPIEGTPYCFSRND
eee-eeeeeeeeeeeeeeeeeeeeeeeeeeeeeeeee---ee-e---e-ee-------ee

SLSSLDFDDDDVDLSREKAELRKAKENKESEAKVTSHTELTSNQQSANKTQAIAKQPINR

--ee-e-eeeeeeeeeeeeeeeeeeeeeeeeeee-eeeeeeeeeeeeeeeee-eeeeeee

GQPKPILQKQSTFPQSSKDIPDRGAATDEKLQNFAIENTPVCFSHNSSLSSLSDIDQENN

eeeee--eeeee-eeeeeeeeeeee-eeeeeee--eee------e-ee-ee-eeeeeeee

NKENEPIKETEPPDSQGEPSKPQASGYAPKSFHVEDTPVCFSRNSSLSSLSIDSEDDLLQ

eeeeeeeeeeeeeeeeeeeeeee-eeeeeeeeeeeeeee--eeeeeeeeee-eeeee--e

ECISSAMPKKKKPSRLKGDNEKHSPRNMGGILGEDLTLDLKDIQRPDSEHGLSPDSENFD

e--eee-eeeeeeeeeeeeeeeeeeeeeeeeeeeeeeee-eeeeeeeeeeeeeee-eeee

WKAIQEGANSIVSSLHQAAAAACLSRQASSDSDSILSLKSGISLGSPFHLTPDQEEKPFT

-e--eee-e--eee-ee-eeeeeeeeeeeeeeeeeee-eeee-eeeeeeeeeeeeeeeee

SNKGPRILKPGEKSTLETKKIESESKGIKGGKKVYKSLITGKVRSNSEISGQMKQPLQAN

eeeeeeeeeeeeeeeeeeeeeeeeeeeeeeeeeeeeeeeeeeeeeeeeeeeeeeeeeeee

MPSISRGRTMIHIPGVRNSSSSTSPVSKKGPPLKTPASKSPSEGQTATTSPRGAKPSVKS

-e---eee-----eeeeeeeeeeeeeeeeeeeeeeeeeeeeeeeeeeee-eeeeeeeeee

ELSPVARQTSQIGGSSKAPSRSGSRDSTPSRPAQQPLSRPIQSPGRNSISPGRNGISPPN
eeeeeeeeeeeeeeeeeeeeeeeeeeeeeeeeeeeeeeeeeeeeeeeeeeeeeeeeeeee

KLSQLPRTSSPSTASTKSSGSGKMSYTSPGRQMSQQNLTKQTGLSKNASSIPRSESASKG

eeeeeeeeeeeeeeeeeeeeeeeeeee-eee-eeeee-eeeeeeeeeeeeeeeeeeeeee

LNQMNNGNGANKKVELSRMSSTKSSGSESDRSERPVLVRQSTFIKEAPSPTLRRKLEESA

eeeeeeeeeeeee-e-ee-eeeeeeeee-eeeeeee--ee-e--ee-eeeeeeeeeeeee

SFESLSPSSRPASPTRSQAQTPVLSPSLPDMSLSTHSSVQAGGWRKLPPNLSPTIEYNDG

eeeeeeeeeeeeeeeeeeeeeeeeeeeeeeee--eee-eeeeeeeeeeeeeeeeeeeeee

RPAKRHDIARSHSESPSRLPINRSGTWKREHSKHSSSLPRVSTWRRTGSSSSILSASSES

eeeeeeeeeeeeeeeeee-eeeeeeeeeeee-eeeee-ee-e-eeeeeeeeeeeeeeeee

SEKAKSEDEKHVNSISGTKQSKENQVSAKGTWRKIKENEFSPTNSTSQTVSSGATNGAES

eee-eeeeeeeeeeeeeeeeeeeeeeeeeee-ee-eeee--eeeeeeeeeeeeeeeeeee

KTLIYQMAPAVSKTEDVWVRIEDCPINNPRSGRSPTGNTPPVIDSVSEKANPNIKDSKDN

eeeeeeeeeeeeeeee-eee-ee-eeeeeeeeee-eeeeeee-eeeeeee-eeeeeeeee

QAKQNVGNGSVPMRTVGLENRLNSFIQVDAPDQKGTEIKPGQNNPVPVSETNESSIVERT

eeeeeeeeee-eeeeeeeeeeeeeeee-eeeeeeeeeeeeeeeee-eeeeeeeeeeeee-

PFSSSSSSKHSSPSGTVAARVTPFNYNPSPRKSSADSTSARPSQIPTPVNNNTKKRDSKT

eeeeeeeeeeeeeeeee-ee-eeee-ee-eeeeeeeeeeeeeee-e-eeeeeeeeeeeee

DSTESSGTQSPKRHSGSYLVTSV
eeeeeeeeeeeeeeee----eee

**Fig I(ii): The result of SCRATCH server of APC protein where the yellow color highlighted region represent OLP, “e” represents surface accessible residue and “-“ represents buried residue.**

**.**

**(iii) MDM2_HUMAN:**

MCNTNMSVPTDGAVTTSQIPASEQETLVRPKPLLLKLLKSVGAQKDTYTMKEVLFYLGQY

eeeeeeeeeeeeeee-ee-eee-eeee-e-eee--e--ee-e-eeee---ee-ee--ee-

IMTKRLYDEKQQHIVYCSNDLLGDLFGVPSFSVKEHRKIYTMIYRNLVVVNQQESSDSGT

-eeee--eeeeee--e-ee-e--e--eeee--eeeeee--e--ee--eeeeeeeeeeeee

SVSENRCHLEGGSDQKDLVQELQEEKPSSSHLVSRPSTSSRRRAISETEENSDELSGERQ

eeeeeee-eeeeeeeeeeeee-eeeeeeeeeeeeeeeeeeeeee-eeeeeeeeeeeeeee

RKRHKSDSISLSFDESLALCVIREICCERSSSSESTGTPSNPDLDAGVSEHSGDWLDQDS

eeeeeeeeeeeeeeee-------e---eeeeeeeeeeeeeeee-eee--eeeee--eee-

VSDQFSVEFEVESLDSEDYSLSEEGQELSDEDDEVYQVTVYQAGESDTDSFEEDPEISLA

eeee-e---e-eeeeeee-e-eeeeeeeeeeeee--e-e--eeeeeeeeeeeeeeeeeee

DYWKCTSCNEMNPPLPSHCNRCWALRENWLPEDKGKDKGEISEKAKLENSTQAEEGFDVP

e--e-eeeeee--eeee--eeee-eeeeeeeeeeeeeeeeeeeeeeeeeeeeeeee-e--

DCKKTIVNDSRESCVEENDDKITQASQSQESEDYSQPSTSSSIIYSSQEDVKEFEREETQ

e-eeeeeeeeee--eeeeeeeeeeeeeeeeeeeeeeeeeeeeeeeeeeee-eeeeeeeee

DKEESVESSLPLNAIEPCVICQGRPKNGCIVHGKTGHLMACFTCAKKLKKRNKPCPVCRQ

eeeeeeeeeeeee-eee-eeeeeee-------eee-------e--ee-eeeeee-eeeee

PIQMIVLTYFP

e-ee----e-e

**Fig I(iii): The result of SCRATCH server of MDM2 protein where the yellow color highlighted region represent OLP, “e” represents surface accessible residue and “-“ represents buried residue.**

**.**
